# Impact of angiotensin-converting enzyme inhibitors and angiotensin receptor blockers on cardiovascular and non-cardiovascular outcomes in patients at high/very-high cardiovascular risk without heart failure: a systematic review and meta-analysis of randomized, double-blind, placebo-controlled trials ^†^

**DOI:** 10.1093/ehjopen/oeag119

**Published:** 2026-07-28

**Authors:** Stefano Masi, Gianluigi Savarese, Nicola Orsini, Martin H Strauss

**Affiliations:** Department of Clinical and Experimental Medicine, University of Pisa, Via Roma, 67, Pisa 56126, Italy; Department of Clinical Science and Education at Södersjukhuset, Karolinska Institutet, Stockholm 171 77, Sweden; Department of Global Public Health, Karolinska Institutet, Norrbackagatan 4, Stockholm 171 76, Sweden; North York General Hospital, University of Toronto, 4001 Leslie St, North York, ON M2K 1E1, Canada

**Keywords:** Angiotensin-converting enzyme inhibitors, Angiotensin receptor blockers, Cardiovascular mortality, Meta-analysis, Placebo-controlled trials

## Abstract

**Aims:**

To assess the impact of angiotensin-converting enzyme inhibitors (ACE-Is) and angiotensin receptor blockers (ARBs) on all-cause and cardiovascular (CV) mortality, as well as CV, cerebrovascular, and renal outcomes in patients at high/very-high CV risk without heart failure by a systematic review and meta-analysis of placebo-controlled, double-blind, randomized trials.

**Methods and results:**

The CENTRAL, ClinicalTrials.gov, Ovid-MEDLINE, Ovid-Embase, Science Citation Index-Expanded, and WHO-ICTRP databases were searched between August and September 2023. Heart failure trials were excluded. The hypothesis of a homogeneous treatment effect between ACE-Is and ARBs was tested with a Cochran’s Q statistic. The protocol for the systematic review was registered on PROSPERO (CRD42023452406) and the study reported as per PRISMA guidelines.

17 trials (9 ACE-Is and 8 ARBs, total of 87 908 participants) were included. The event rates for all-cause mortality, CV mortality, and major CV events (MACEs) did not differ in the parallel placebo arms of ACE-I and ARB trials; however, compared to placebo, ACE-Is reduced the rate of CV mortality [hazard ratio (HR) 0.88; 95% confidence interval (CI) 0.78–0.99], all-cause mortality (HR 0.92; 95% CI 0.85–0.99) and myocardial infarction (HR 0.83; 95% CI 0.73–0.94), while ARBs did not. The rates of HF, stroke, and MACEs were significantly reduced by both drug classes. Angiotensin-converting enzyme inhibitors induced a greater reduction of CV mortality than ARBs (*Q* = 4.59; *P*-value = 0.03).

**Conclusion:**

In patients at high/very-high CV risk, ACE-Is were more protective against CV mortality than ARBs, supporting their preferential use for CV protection.

## Graphical Abstract

**Graphical Abstract:**
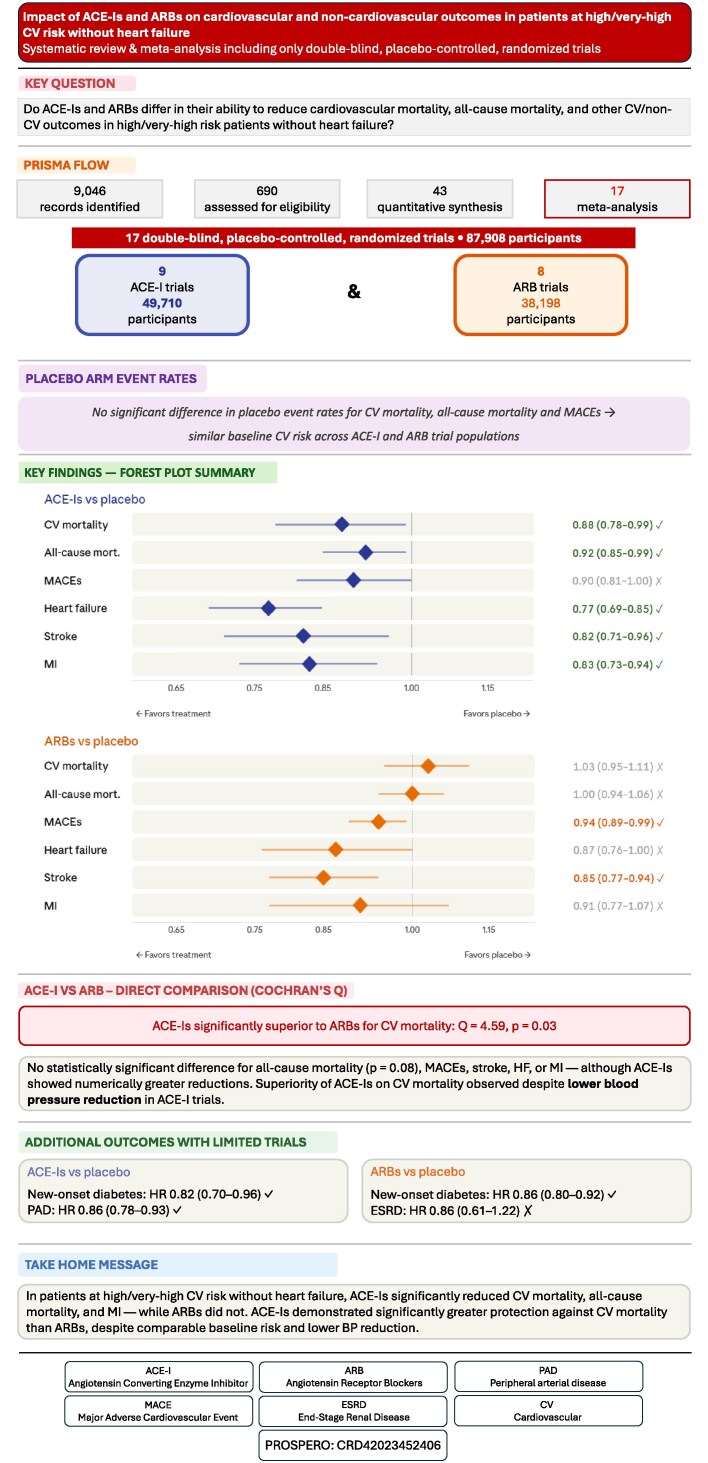

## Introduction

Cardiovascular, cerebrovascular, and kidney diseases remain highly prevalent conditions worldwide and are major contributors to disability-adjusted life years.^1^ Angiotensin-converting enzyme inhibitors (ACE-Is) and angiotensin II receptor blockers (ARBs) effectively reduce renin–angiotensin–aldosterone system (RAAS) signalling and represent the cornerstone of cardiovascular (CV), kidney, and cerebrovascular disease protection. While some guidelines recommend ACE-Is and ARBs as interchangeable options,^2–5^ their differing pharmacodynamic profiles suggest that they may exert distinct CV and renal protective effects, and an increasing number of guidelines suggest using ACE-Is as the first-line therapy in patients at high/very high CV risk, leaving ARBs as the second-line treatment, limited to the ACE-I intolerant patients.^6–10^ Previous meta-analyses have compared the relative benefits of ACE-Is and ARBs in preventing CV, cerebrovascular, and renal outcomes.^11–15^ However, only a few head-to-head trials have directly evaluated the differential effects of these two drug classes on such outcomes, making them difficult to compare.^16^ Moreover, results of meta-analyses of randomized controlled trials (RCTs) that separately assessed the protective effects of ACE-Is and ARBs against placebo or other active comparators on major CV outcomes have been differently interpreted.^11,13^ These inconsistencies are fuelled by differences in study design (e.g. double-blind vs. open-label trials), outcomes measured [e.g. major CV events (MACEs), all-cause mortality, or CV mortality], and patient populations [e.g. those with or without heart failure (HF)] of different meta-analyses. Consequently, it remains difficult comprehensively understand the effects of ACE-Is and ARBs on the full range of adverse outcomes associated with arterial hypertension.

To address these challenges, we conducted a systematic review and meta-analysis with the primary objective to evaluate the effects of ACE-Is and ARBs on a broad spectrum of CV and renal outcomes in patients at high/very high CV risk, regardless of the presence of arterial hypertension. Additionally, as most of the CV and nephroprotective effects of ACE-I and ARBs might depend on blood pressure (BP) lowering, we excluded studies involving patients with HF, as outcomes in such cases may depend on factors beyond BP control. In a second part of our study, we used Cochran’s Q statistic to directly compare the treatment effects of ACE-Is and ARBs on each outcome (secondary objective). In an attempt to avoid the limitations of previous reports, we exclusively included double-blind, randomized, placebo-controlled trials in the meta-analysis. This decision was motivated by the aim of providing more consistent estimates of the potential effects of ACE-Is and ARBs on each outcome, which could be confounded in trials that include active drugs in the control group.

## Methods

This study was designed according to the PRISMA (Preferred Reporting Items for Systematic reviews and Meta-Analyses), statement and the systematic review was registered in PROSPERO (CRD42023452406).

### Systematic literature search

Six different databases were searched between August 2023 and September 2023, without date, language, document type, or publication status restrictions: The Cochrane Central Register of Controlled Trials (CENTRAL), ClinicalTrials.gov, Ovid MEDLINE, Ovid Embase, Science Citation Index-Expanded, and WHO ICTRP. Validated search filters were used to retrieve RCTs from Embase^17^ and MEDLINE.^18^ We used reference lists of the retrieved articles to identify additional eligible studies. The rationale for keyword selection is reported in the Supplementary methods and Supplementary material online, *Table S1*.

### Study selection, data extraction, synthesis, and quality assessment

Studies were included if they were double-blind, randomized controlled clinical trials comparing ACE-Is or ARBs with placebo. Only trials reporting on all-cause mortality, with a prespecified duration of follow-up of ≥1 year, ≥100 participants in each study arm, ≥1000 person-years follow-up, with the outcome of interest occurring in ≥10 participants in each arm were considered for the analysis. Open-label studies (even if they are randomized or have blinded assessment of outcomes) or trials in which ARBs could be added to an initial regimen of ACE-Is or vice versa were excluded. Heart failure trials and those evaluating initial dual RAAS inhibitor-based combinations at baseline were also excluded. Notably, HF trials were excluded because CV and renal outcomes in these patients may depend more on haemodynamic factors—which often preclude full implementation of RAAS inhibitor therapy—than on the specific class of RAAS inhibitor, making their results not directly comparable with those of other conditions. Furthermore, most patients with HF previously treated with RAAS inhibitors (i.e. those with reduced ejection fraction and a substantial proportion of those with preserved ejection fraction) now have specific indications for sacubitril/valsartan combination therapy, rendering any potential differences observed between RAAS inhibitor classes less relevant to daily clinical practice.

**Figure 1:**
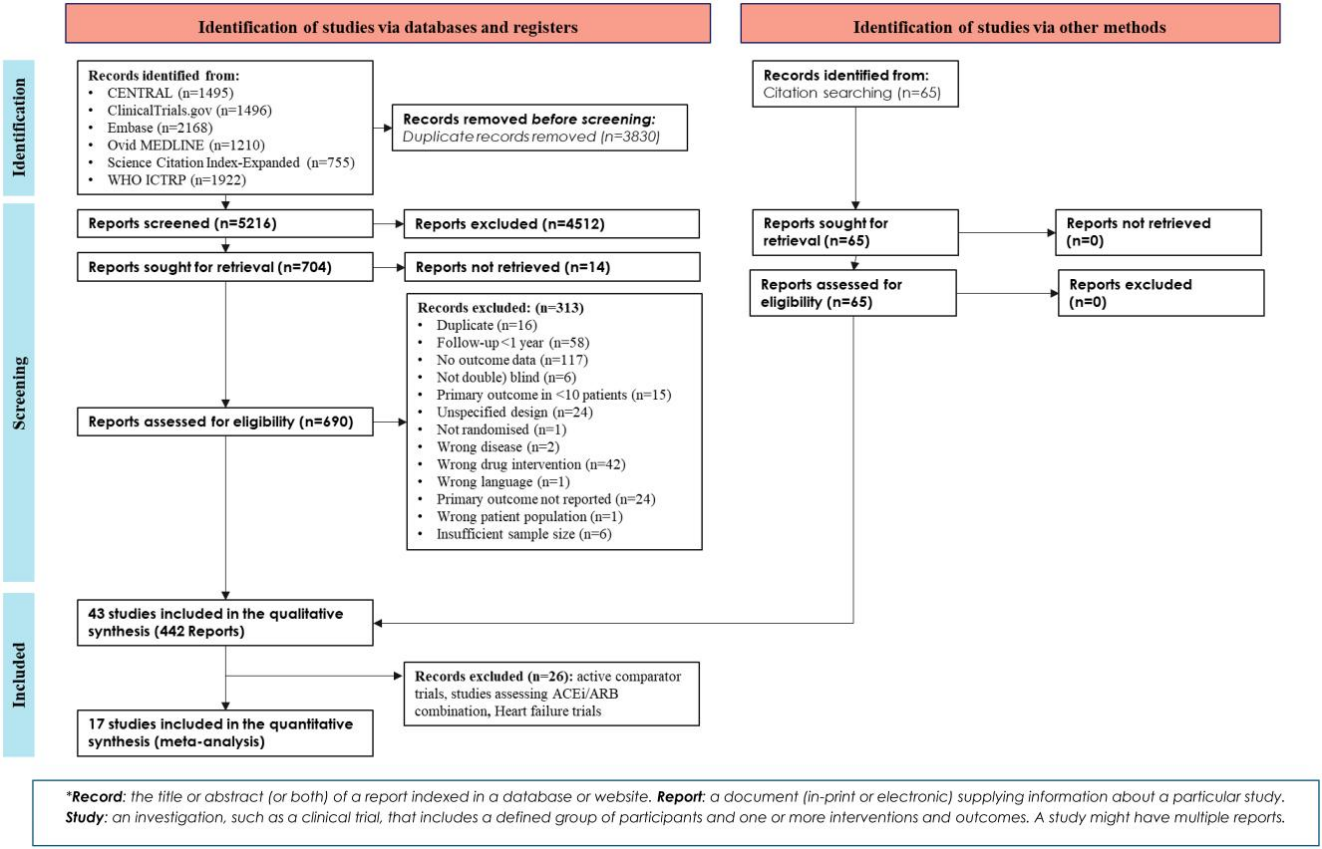
PRISMA flowchart for study selection.

Only trials that assessed ACE-Is or ARBs vs. placebo were finally considered in the meta-analysis. *Figure 1* reports the PRISMA flowchart for the study selection.

Further details on the steps followed for study selection are provided in the Supplementary file, which also reports the standardized pre-piloted data extraction form used to extract the trial data (Supplementary material online, *Figure S1*).

Log hazard ratios (or log rate ratios) and standard error were extracted for all outcomes. When published relative risk reductions were available, the hazard ratio and its standard error were estimated using the follow-up duration and event probability in each arm, based on an exponential survival model (see Supplementary methods).

The risk of bias was assessed using the Cochrane Collaboration’s Risk of Bias tool (version 1).^19^ The certainty of evidence was rated according to the GRADE system.^20,21^

### Statistical analyses

Study participants’ characteristics and outcomes were summarized in tables using descriptive statistics. The outcomes considered in the meta-analysis were CV mortality, all-cause mortality, MACEs, stroke, myocardial infarction (MI), incident HF, end-stage renal failure, peripheral vascular disease, and new-onset type 2 diabetes. We retained the definition of MACEs reported in each original trial to facilitate transparent reporting and exploration of potential heterogeneity, as previously suggested.^22^ A detailed description of the statistical analyses is provided in the Supplementary file. To assess the robustness of the main results, a leave-one-out meta-analysis was conducted by iteratively repeating the meta-analysis while excluding one study at a time.

## Results

Baseline characteristics of the placebo-controlled trials included in the meta-analysis are reported in *Tables 1 and 2*. The total number of patients recruited in the 17 trials included in the final meta-analysis was 87 908, of whom 49 710 were in ACE inhibitor trials and 38 198 in ARB trials. The overall mean age of subjects was similar, corresponding to 63 ± 5 years in ACE-Is trials and 64 ± 6 years in ARB trials. The average length of follow-up was not different between ACE-I (3.3 ± 1.3 years) and ARB (3.9 ± 0.8 years) trials. The average systolic and diastolic BP values were higher in the ARB than those in the ACE-I trials, but these values recorded at the end of the follow-up were similar for both the trials, suggesting a more substantial BP reduction in ARB than in ACE-I trials (*Tables 1 and 2*). The proportion of MACEs in the placebo groups was 17% in ACE-I trials over a mean follow-up of 3.3 years and 20% in ARB trials over 3.8 years. This rate of events was consistent with populations at high/very high CV risk. Importantly, the summary event rates for MACEs, CV mortality, and all-cause mortality in the placebo group of both ACE-I and ARB trials were not significantly different between the two classes of drugs (*P*-values very close to 1, Supplementary material online, *Figure S2A*–*C*). These results suggest a comparable overall CV risk profile among populations included in the ACE-I and ARB trials.

**Table 1:** Demographic and clinical characteristics of the population included in ACE-I trials

ACE-I trials	Study arm	Number of subjects	Age (years)	Systolic BP at baseline (mmHg)	Diastolic BP at baseline (mmHg)	Systolic BP at the end of follow-up (mmHg)	Diastolic BP at the end of follow-up (mmHg)	Sex (% Male)	BMI (kg/m^2^)	Hypertension (%)	Diabetes (%)	Average follow-up (years)
DIABHYCAR^23^	Ramipril	2456	65	146	82	142	80	70	29	70	100	4
	Placebo	2456	65	145	82	143	80	70	29	70	100	
DREAM^24^	Ramipril	2623	55	136	83	128	78	40	31	43	0	3
	Placebo	2646	55	136	83	132	80	41	31	44	0	
EUROPA^25^	Perindopril	6110	60	137	82	127	78	85	28	27	12	4.2
	Placebo	6108	60	137	82	132	80	85	28	27	13	
HOPE^26^	Ramipril	4645	66	139	79	136	76	72	28	48	39	4.5
	Placebo	4652	66	139	79	139	77	74	28	46	38	
PART-2^27^	Ramipril	308	60	133	79	127	74	82	—	100	3	2
	Placebo	309	61	133	79	132	78	82	—	100	3	
PEACE^28^	Trandolapril	4158	64	134	78	130	74	81	—	46	18	4.8
	Placebo	4132	64	133	78	132	76	83	—	45	16	
PREAMI^29^	Perindopril	631	72	126	74	134	78	65	27	59	25	1
	Placebo	621	73	125	74	137	79	65	26	57	23	
PROGRESS^30^	Perindopril	3051	64	147	86	—	—	70	—	48	13	3.9
	Placebo	3054	64	147	86	—	—	70	—	48	12	
QUIET^31^	Quinapril	872	58	123	74	—	—	82	26	48	14	2
	Placebo	878	58	123	74	—	—	81		47	16	
SUMMARY	ACE-I arm	24 854	62.7	136	80	132	77	72	28	54	25	3.3
	Placebo arm	24 856	62.9	135	80	135	79	72	28	54	25	
	Total number patients in ACE-I trials	49 710	62.8	135	80	134	78	72	28. 3	54	25	

ACE-I, angiotensin-converting enzyme inhibitor; BMI, body mass index; BP, blood pressure; DIABHYCAR, DIABetes, HYpertension, CArdiovascular events and Ramipril; DREAM, diabetes reduction approaches with ramipril and rosiglitazone medications; EUROPA, European trial on reduction of cardiac events with perindopril in stable coronary artery disease; HOPE, heart outcomes prevention evaluation; PART-2, prevention of atherosclerosis with ramipril trial; PEACE, prevention of events with angiotensin-converting enzyme inhibition; PREAMI, perindopril and remodeling in elderly with acute myocardial infarction; PROGRESS, perindopril protection against recurrent stroke study; QUIET, QUinapril Ischemic Event Trial.

**Table 2:** Demographic and clinical characteristics of the population included in ARB trials

ARB trials	Study arm	Number of subjects	Age (years)	Systolic BP at baseline (mmHg)	Diastolic BP at baseline (mmHg)	Final systolic BP (mmHg)	Final diastolic BP (mmHg)	Sex (% Male)	BMI (kg/m^2^)	Hypertension (%)	Diabetes (%)	Average follow-up (years)
ACTIVE-I^32^	Irbesartan	4518	70	138	83	132	78	61	29	88	20	4.1
	Placebo	4498	70	138	82	135	80	61	29	88	20	
DIRECT-Protect-2^33^	Candesartan	951	57	—	—	—	—	49	29	62	100	4.7
	Placebo	954	57	—	—	—	—	51	29	62	100	
IDNT^34^	Irbesartan	579	59	160	87	140	77	65	31	100	100	2.6
	Placebo	569	58	158	87	144	80	71	31	100	100	
NAVIGATOR^35^	Valsartan	4631	64	139	82	133	78	50	30	77	0	5.0
	Placebo	4675	64	140	83	136	80	49	31	77	0	
RENAAL^36^	Losartan	751	60	152	82	140	74	62	30	92	100	3.4
	Placebo	762	60	153	82	142	74	65	29	95	100	
ROADMAP^37^	Olmesartan	2232	58	137	81	126	74	47	31	75	100	3.2
	Placebo	2215	58	136	80	129	76	45	31	75	100	
SCOPE^38^	Candesartan	2477	76	166	90	145	80	35	27	100	13	3.7
	Placebo	2460	76	166	90	149	82	36	27	100	12	
TRANSCEND^39^	Telmisartan	2954	67	141	82	—	—	57	28	77	36	4.7
	Placebo	2972	66	141	82	—	—	57	28	76%	36	
SUMMARY	ARB arm	19 093	64	148	84	136	77	53%	29	87%	59	3.9
	Placebo arm	19 105	64	147	84	139	79	54	29	87	59	
	Total number patients in ARB trials	38 198	64	147	84	138	78	54	29	84	59	

ACTIVE-I, atrial fibrillation clopidogrel trial with irbesartan for prevention of vascular events; ARB, angiotensin receptor blocker; BMI, body mass index; BP, blood pressure; DIRECT-Protect-2, diabetic retinopathy candesartan trials – Protect 2; IDNT, irbesartan diabetic nephropathy trial (also reported as Irbesartan Type II Diabetic Nephropathy Trial); NAVIGATOR, nateglinide and valsartan in impaired glucose tolerance outcomes research; RENAAL, reduction of endpoints in NIDDM with the angiotensin II antagonist losartan; ROADMAP, randomized olmesartan and diabetes microalbuminuria prevention; SCOPE, study on cognition and prognosis in the elderly; TRANSCEND, telmisartan randomised assessment study in ACE intolerant subjects with cardiovascular disease.

### Primary objective

#### Effects of ACE-Is vs. placebo

**Figure 2:**
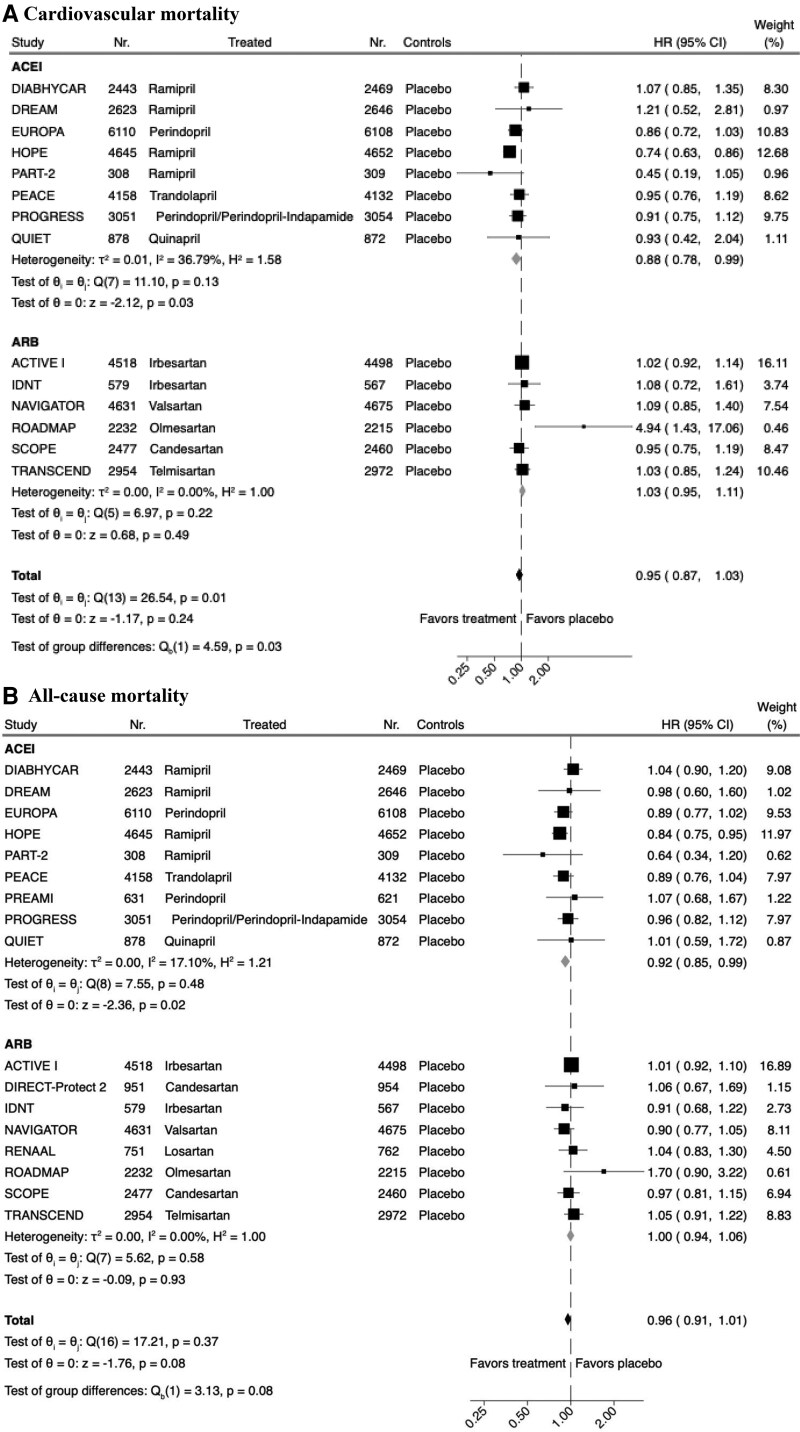

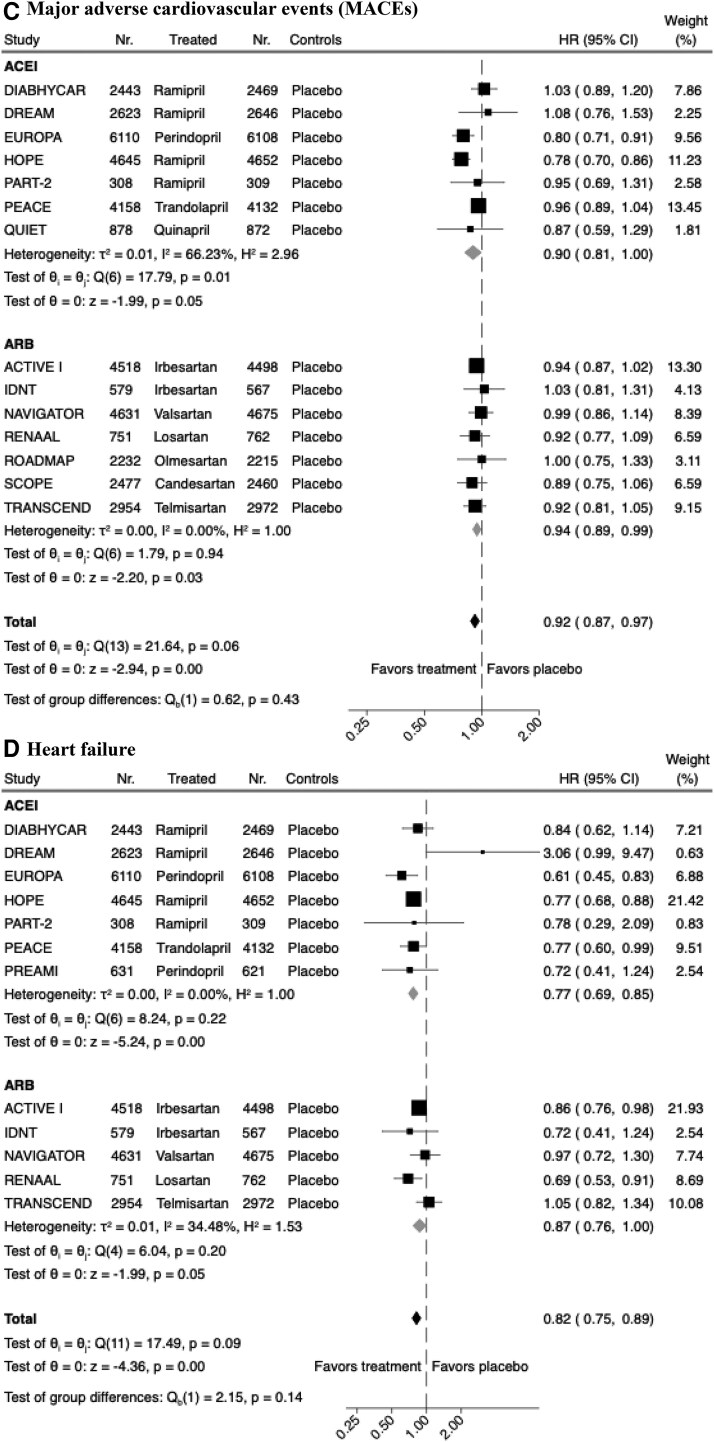

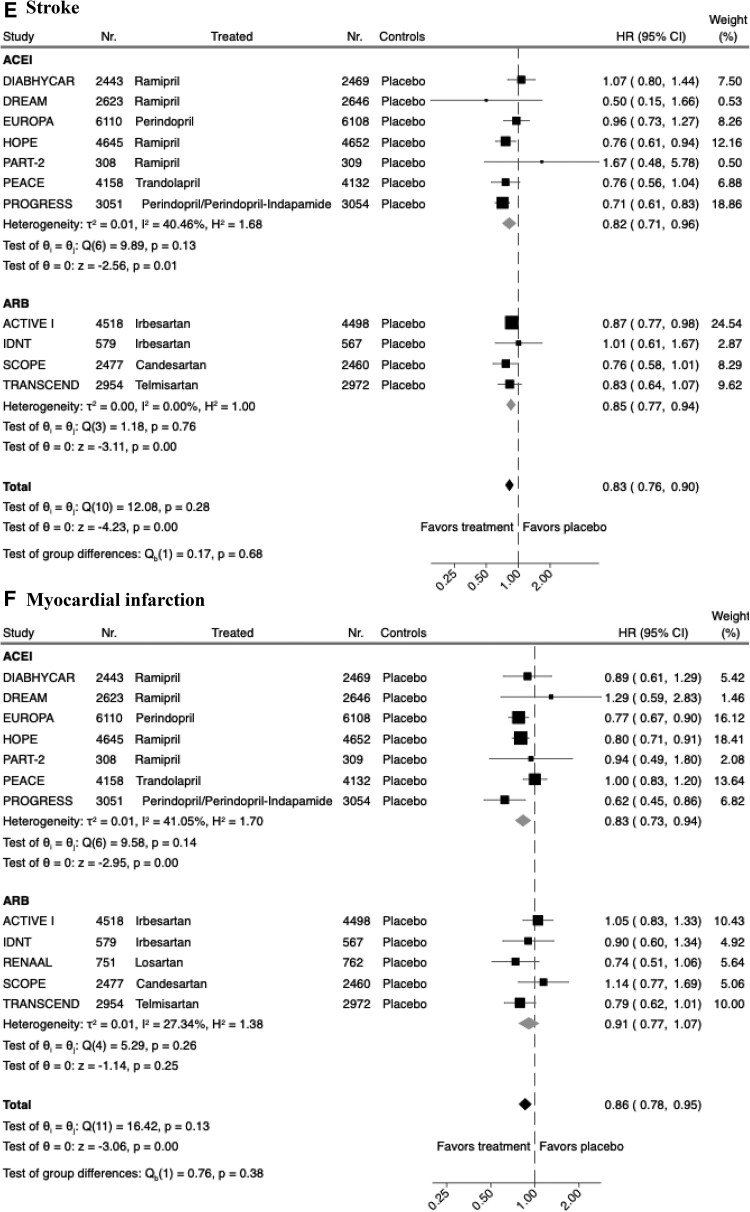
The impact of ACE-Is and ARBs on the outcomes of interest against placebo: (*A*) cardiovascular mortality, (*B*) all-cause mortality, (*C*) MACEs, (*D*) heart failure, (*E*) stroke, (*F*) myocardial infarction. ACE-I, angiotensin-converting enzyme inhibitor; ARB, angiotensin receptor blockers; MACE, major adverse cardiovascular event.

ACE-Is vs. placebo led to 12% reduction in CV mortality [hazard ratio (HR): 0.88, 95% confidence interval (CI): 0.78–0.99], heterogeneity *I*^2^ = 36.79%) (*Figure 2A*), 8% reduction in the risk of all-cause mortality (HR: 0.92, 95%CI: 0.85–0.99], heterogeneity I^2^ = 17.10%) (*Figure 2B*), 10% reduction in the risk of MACEs (HR: 0.90, 95% CI: 0.81–1.00, heterogeneity *I*^2^ = 66.23%) (*Figure 2C*), 23% reduction in the risk of HF (HR: 0.77, 95% CI: 0.69–0.85, heterogeneity *I*^2^ = 0.00%) (*Figure 2D*), 18% reduction in the risk of stroke (HR: 0.82, 95% CI: 0.71–0.96, heterogeneity *I*^2^ = 40.46%) (*Figure 2E*), and 17% reduction in the risk of MI (HR: 0.83, 95% CI: 0.73–0.94, heterogeneity *I*^2^ = 41.05%) (*Figure 2F*). Compared to placebo, ACE-Is also significantly reduced the risk of new-onset diabetes (HR: 0.82, 95% CI: 0.70–0.96, heterogeneity *I*^2^ = 62.88%) and peripheral artery disease (HR: 0.86, 95% CI: 0.78–0.93, heterogeneity *I*^2^ = 0.00%), but for these outcomes and the risk of end-stage renal disease, there was a limited number of trials available for the meta-analysis (three for new-onset diabetes, three for peripheral artery disease, and one for end-stage renal disease) (data not shown).

#### Effects of ARBs vs. placebo

When compared with placebo, ARBs did not cause a significant reduction in the risk of CV mortality (HR: 1.03, 95% CI: 0.95–1.11, heterogeneity *I*^2^ = 0.00%) (*Figure 2A*), all-cause mortality (HR: 1.00, 95% CI: 0.94–1.06, heterogeneity *I*^2^ = 0.00%) (*Figure 2B*), and MI (HR: 0.91, 95% CI: 0.77–1.07, heterogeneity *I*^2^ = 27.34%) (*Figure 2F*). A significant reduction in the risk of MACEs (HR: 0.94, 95% CI: 0.89–0.99, heterogeneity *I*^2^ = 0.00%) (*Figure 2C*), HF (HR: 0.87, 95% CI: 0.76–1.00, heterogeneity *I*^2^ = 34.48%) (*Figure 2D*), and stroke (HR: 0.85, 95% CI: 0.77–0.94, heterogeneity *I*^2^ = 0.00%) (*Figure 2E*) was observed as compared to placebo. Angiotensin receptor blockers also significantly reduced the risk of diabetes (HR: 0.86, 95% CI: 0.80–0.92, heterogeneity *I*^2^ = 0.00%) but not of end-stage renal disease (HR: 0.86, 95% CI: 0.61–1.22, heterogeneity *I*^2^ = 73.77%), although for these outcomes and for the risk of peripheral arterial disease, the number of trials available for the meta-analysis was limited (two for diabetes, three for end-stage renal disease, and only one for peripheral artery disease) (data not shown).

### Secondary objective

The test for group difference revealed that the magnitude of CV death rate reduction with ACE-Is was greater than with ARBs (*Q* = 4.59; *P*-value = 0.03). We did not observe other significant differences between ACE-Is and ARBs for the other outcomes.

### Risk of bias and GRADE

Supplementary material online, *Tables S2* and *S3* provide an assessment of the risk of bias and the certainty of evidence using the grading of recommendations assessment, development, and evaluation (GRADE) system. Among ACE-Is trials, a mix of ‘Low’ and ‘Unclear’ ratings dominate, with multiple studies like European trial on reduction of cardiac events with perindopril in stable coronary artery disease (EUROPA), heart outcomes prevention evaluation (HOPE), and prevention of events with angiotensin-converting enzyme inhibition (PEACE) showing uncertainty in random sequence generation and allocation concealment. The PROGRESS trial stands out with consistently low risks across all domains, making it one of the most robust studies in this group. However, other trials, such as DIABetes, HYpertension, CArdiovascular events and Ramipril (DIAHBYCAR) and perindopril and remodeling in elderly with acute myocardial infarction (PREAMI), display uncertainty in multiple categories, indicating a need for cautious interpretation. In the ARB category, the irbesartan diabetic nephropathy trial (also reported as irbesartan Type II diabetic nephropathy trial) (IDNT) trial shows a ‘High’ risk in random sequence generation and ‘Unclear’ in other areas. Meanwhile, studies like atrial fibrillation clopidogrel trial with irbesartan for prevention of vascular events (ACTIVE I) and study on cognition and prognosis in the elderly (SCOPE) exhibit ‘Unclear’ biases in certain aspects. Overall, while many studies demonstrate strong methodological rigour, areas of uncertainty suggest potential bias risks in some trials.

Regarding the GRADE, the level of evidence for ACE-I and ARBs for most outcome was moderate to high, with a poorer score for ARBs than for ACE-Is (see Supplementary material online, *Table S3*).

### Sensitivity analyses

The ‘leave-one-out’ analysis reported consistent results of the outcome analysis for ACE-Is and ARBs regardless of the excluded trials (see Supplementary material online, *Figure S3*).

## Discussion

Placebo-controlled trials are the ‘gold standard’ for evaluating drug efficacy and safety. When analysed separately for our primary objective, ACE-Is were associated with a significant reduction in the risk of CV mortality, all-cause mortality, and MI compared to placebo, whereas ARBs did not show such benefits. When the two drug classes were directly compared in terms of their relative effects vs. placebo on the outcomes included in the meta-analysis, ACE-Is produced a significantly greater reduction in CV mortality risk than ARBs. These findings were observed despite a more modest reduction in BP in the ACE-I trials compared to the ARB trials, and despite a comparable incidence of all-cause mortality, CV mortality, and MACEs in the placebo arms of both trial groups, suggesting similar baseline risk profiles across populations. In summary, this meta-analysis of double-blind, randomized, placebo-controlled trials assessing the effects of ACE-Is and ARBs on CV outcomes indicates that ACE-Is offer broader and more consistent protection than ARBs. Notably, the superiority of ACE-Is in reducing CV mortality does not appear to be attributable to differences in baseline CV risk or to the degree of BP lowering achieved during the trials.

There is biological plausibility supporting results of our meta-analyses. Although both ACE-Is and ARBs target the RAAS, each has its unique modes of action. ACE-Is suppress angiotensin II (ANG II) levels and attenuate both the indirect effects of ANG II and its direct tissue toxicity. ACE-Is also prevent the breakdown of bradykinin, which has well known cardioprotective effects.^40^ Angiotensin receptor blockers, in contrast, do not inhibit the breakdown of bradykinin levels and, while blocking the negative effects of ANG II on its AT_1_ receptor, they induce a rebound and sustained increase in circulating ANG II levels.^40^ This ‘rebound’ might harm vascular homeostasis, as ANG II might stimulate other receptors, such as the AT_2_ receptors, potentially impairing vascular physiology by promoting the acquisition of a pro-atherosclerotic vascular phenotype.^40^ These pharmacodynamic considerations might explain the protection against the risk of MI obtained with ACE-Is but not ARBs when compared with placebo, as well as the greater effect of ACE-Is than ARBs on the protection from CV mortality when the two classes of drugs are directly compared.

While the results of our meta-analysis are consistent with the work of others, several strengths differentiate our study from previously published papers on the same topic. Savarese *et al*. performed a similar meta-analysis, including only placebo-controlled and excluding HF trials. The results documented that, as compared to placebo, both ACE-Is and ARBs reduced the risk of MACEs, stroke, and new-onset diabetes, but only ACE-Is reduced the risk of all-cause mortality, MI, and new-onset HF.^13^ By restricting the analysis to trials with a minimum number of events in each treatment arm, we were much more selective in our inclusion criteria, leading to a much lower number of trials included in the current meta-analysis. This might explain the lack of some significant differences as observed by Savarese *et al*. An additional strength of the current meta-analysis, compared to the study by Savarese *et al*., is its broader assessment of outcomes. In addition to CV and cerebrovascular disease, it also includes peripheral arterial disease and end-stage renal failure. This expanded scope is important, as it allowed us to highlight the limited evidence from placebo-controlled trials supporting the effects of ACE-Is and ARBs on these outcomes—underscoring the need for future research to address this evidence gap.

A previous meta-analysis by van Vark *et al*. vs. all comparators documented that ACE-Is confer superior protection from all-cause mortality than ARBs.^15^ Although a borderline trend towards a greater reduction from all-cause mortality with ACE-Is vs. ARBs emerged also in our meta-analysis, the test for group difference did not reach statistical significance. Among the reasons accounting for this difference, it should be noted that in the van Vark meta-analysis, trials were included only in the case when ≥2/3 of the subjects had arterial hypertension at baseline, while we included trials that enrolled patients at high/very high CV risk, regardless of the percentage of patients with hypertension at baseline. Furthermore, compared to van Vark’s meta-analysis, we did not include trials comparing ACE-Is or ARBs vs. active comparators. Again, these additional filters resulted in fewer trials available for our meta-analysis, potentially explaining the borderline but non-significant trend in favour of a superior protection from ACE-Is vs. ARBs against all-cause mortality.

Regarding the risk of MI, our findings are consistent with those reported by Bangalore *et al*., who showed that ARBs were not associated with a significant reduction in MI risk (relative risk: 0.99; 95% CI: 0.92–1.07) compared to control treatment. In the same meta-analysis, ARB therapy also failed to significantly reduce the risk of all-cause mortality, CV mortality, or angina pectoris, results that align with our own.^11,16^ The heterogeneity of control treatments in the trials included in that meta-analysis (ranging from placebo to various active drugs) raises the possibility that the lack of observed benefit with ARBs may be partly explained by the differing impacts of the comparators across studies. In contrast, our analysis is less affected by these limitations, as it exclusively includes placebo-controlled trials, thereby offering a more consistent and unbiased design for evaluating the efficacy of ARBs.

When directly comparing the degree of protection against the different outcomes offered by the two drug classes vs. placebo, ACE inhibitors demonstrated a superior effect in reducing the risk of CV mortality compared to ARBs. Although some have suggested that all-cause mortality represents the most robust outcome for CV outcome trials, it may be dominated by unrelated causes of death. Therefore, when assessing the impact of CV protective therapies, all-cause mortality may be less sensitive and specific in evaluating their efficacy compared to CV mortality. Similar to our findings, Bangalore *et al*. documented that ACE-Is but not ARBs reduced the rate of all-cause mortality, CV death, and MI compared to placebo in a larger meta-analysis that was not limited to double-blind controlled trials.^16^ However, after performing a meta-regression and a sensitivity analysis that excluded trials published before 2000, it was concluded that the difference between ACE-Is and ARBs compared to placebo was dependent on a higher placebo event rate in the ACE-I trials for the outcome of all-cause mortality, CV death, and MI. We now show that when including only placebo-controlled, double-blind trials, the rates of CV mortality, all-cause mortality, and MACEs are similar in the placebo groups of ACE-I and ARB trials. This suggests that despite some differences in the prevalence of individual risk factors across ACE-I and ARB trials, the populations included in the two groups of trials were at similar risk of mortality and CV events at baseline. This provides robust evidence that patients enrolled in ACE-I and ARB trials had a similar CV risk profile, making any stratification of the studies based on an arbitrary publication-year cutoff unnecessary, also considering it would not be supported by a clear rationale. In addition, we document that the benefits obtained with the use of ACE-Is against the risk of CV mortality are evident even in the presence of a lower BP reduction than the one observed in ARB studies, suggesting that the protection against these outcomes conferred by ACE-Is might be partially independent of the BP reduction. A similar result was reported by a study from the Blood Pressure Lowering Treatment Trialists’ Collaboration, showing that ACE-Is were superior to ARBs in protecting from the risk of coronary artery disease, and that the authors concluded that benefit of ACEI was ‘above’ and independent of the effects of BP lowering.^41^

Although head-to-head trials comparing ACE-Is vs. ARBs represent the most appropriate study design to assess the potential differences in the cardioprotective effects of these classes of drugs, there are few such trials. Bangalore *et al*. previously attempted to perform a meta-analysis that included only head-to-head trials comparing ACE-Is vs. ARBs. Still, they were able to retrieve only eight trials for a total of 22 543 patients.^16^ Remarkably, 76% of these patients were from the ONTARGET trial,^42^ which accounted for 98% of all the deaths. This disproportionally increased the weight of the ongoing telmisartan alone and in combination with ramipril global endpoint trial (ONTARGET) on the final results of the meta-analysis. Considering this issue and to avoid the same risk, we performed a leave-one-out analysis showing that our results are robust and not influenced by a single trial. Such an analysis also provides evidence that the date of publication of the different trials is unlikely to modify our results.

Our results might be viewed as discrepant from those observed in the only large RCT directly comparing ACE-Is vs. ARBs, the ONTARGET trial. Indeed, in this large trial, enrolling patients with very high CV risk due to established coronary, peripheral, or cerebrovascular disease or diabetes with end-organ damage, the authors suggested that there was no statistical difference between telmisartan and ramipril in the rate of the primary study outcome.^42^ However, the trial design of ONTARGET was to evaluate for the statistical superiority of telmisartan to ramipril, which it was not. As stated in the methodology of ONTARGET, there was also an additional secondary analysis for ‘statistical non-inferiority’, proving that telmisartan could retain as little as 50% of the risk reduction of ramipril even despite similar event rates.^42^ As ONTARGET was not designed or powered to test for statistical equivalence, it is impossible to prove equivalence, resulting in a second-line indication for telmisartan in ACE-I intolerant patients, as defined by the Food and Drug Administration.

The clinical implications of our meta-analysis might be relevant. When prescribing CV prevention therapies, clinicians cannot predict which specific event a patient may face, making it essential to choose drugs that offer broad protection across outcomes. Our meta-analysis suggests that, when compared with placebo, ACE-Is provide protection against a wider number of outcomes and a superior protection against CV mortality than ARBs. No outcome showed a protective effect of ARBs over placebo that was not also observed with ACE-Is. This provides clinicians with compelling evidence as to which class of RAAS inhibitors may offer wider benefits and therefore should be preferred as initial therapy. Unlike prior meta-analyses, the trials included in our study enrolled patients with a wide range of clinical conditions, including chronic kidney disease, hypertension, and diabetes. Comparing the effects of different RAAS blockers across such varied populations is important, as it enables the generation of evidence more representative of real-world clinical practice, where hypertension often coexists with other diseases. This approach enhances the clinical relevance of our findings. A further strength of our meta-analysis is the inclusion of over 80 000 participants from numerous RCTs, which reduces the likelihood that the findings are influenced by chance or by any single outlier study. The robustness of our findings is further confirmed by the leave-one-out sensitivity analysis, showing consistent results regardless of which study was removed. Moreover, by including only placebo-controlled trials, we reduced heterogeneity in the estimation of the ACE-I and ARB effect on the outcome potentially deriving from the inclusion of trials with heterogeneous therapies delivered in the control group. These methodological strengths strongly support the reliability of our conclusions. Our study also has some limitations. The trials included in the meta-analysis were conducted before the availability of sodium-glucose cotransporter-2 inhibitors and glucagon-like peptide-1 receptor agonists that are known to have a potential complementary action with renin-angiotensin.aldosterone system inhibitors (RAASi) and might substantially influence the risk of some outcomes included in the meta-analysis, such as HF, end-stage renal failure, and new onset of diabetes. We could not perform an individual patient data meta-analysis, making it more difficult to control for confounding factors or to perform dedicated subgroup analyses. The lack of individual participant data also prevented comparison of background therapy between ACE inhibitor and ARB trials. Although this limitation is common to all meta-analyses on this topic,^11,13–16^ it may have contributed to between-study heterogeneity. However, it should be emphasized that our findings largely confirm the results of the meta-analyses from the Blood Pressure Lowering Treatment Trialists’ Collaboration.^41^ By restricting inclusion to trials with the most rigorous study designs, the number of studies available for our meta-analysis was limited, potentially reducing our ability to detect differences previously reported between ACE-Is and ARBs for certain outcomes (e.g. all-cause mortality). All-cause mortality was the most consistently reported outcome across trials, whereas CV mortality and other outcomes were less consistently reported, further limiting the ability to directly compare the effects of ACE-Is and ARBs on them. Finally, some expert opinion papers have proposed that ARBs should be considered first-line therapy in hypertension.^43^ However, it should be emphasized that this position is not supported by clear evidence demonstrating the superiority of ARBs over ACE-Is in randomized, double-blind clinical trials. This is reflected in meta-analyses based on such trials. Indeed, while several meta-analyses suggest a potential superiority of ACE-Is over ARBs in improving certain CV outcomes,^13–15,41^ the opposite finding (namely, a superiority of ARBs over ACE-Is) has not been consistently reported in meta-analyses of RCTs. Some observational and registry-based studies have suggested a possible advantage of ARBs^44^; however, these findings are likely influenced by residual confounding and bias and therefore cannot be considered as robust as evidence derived from RCTs. Therefore, while we acknowledge previous expert opinions and agree that ARBs are an important and often very well-tolerated option, the current evidence base remains insufficient to support their preferential use as first-line therapy in high-risk patients.

## Conclusions

When selecting placebo-controlled, double-blind, randomized clinical trials (a design that is considered the gold standard when planning and conducting clinical trials) including patients at high/very high CV risk, ACE-Is are likely to confer greater protection than ARBs against CV mortality. This difference is unlikely to be related to a different baseline risk of the population included in ACE-I and ARB trials. Our results support the increasing number of guidelines suggesting the use of ACE-I as first-line therapy in patients at high/very high CV risk, leaving ARB as second-line treatment, limited to ACE-I intolerants.^6–10^ Further double-blind, placebo-controlled, randomized trials are necessary to confirm the impact of RAASi on the risk of new onset type 2 diabetes, peripheral arterial disease, and end-stage renal failure.

## Supplementary Material

oeag119_Supplementary_Data

## Data Availability

The data underlying this article will be shared on reasonable request to the corresponding author.
