# Supplementary material for: Impact of angiotensin-converting enzyme inhibitors and angiotensin receptor blockers on cardiovascular and non-cardiovascular outcomes in patients at high/very-high cardiovascular risk without heart failure: a systematic review and meta-analysis of randomized, double-blind, placebo-controlled trials
Source: Eur Heart J Open. 2026 Jul 28;6(4):oeag119. doi: 10.1093/ehjopen/oeag119 (PMC13409322; doi:10.1093/ehjopen/oeag119)

**SUPPLEMENTARY**

**SUPPLEMENTARY METHODS**

**Systematic literature search**

Keywords were collected through previous opinion papers written by internationally recognised experts in the field, literature review, controlled vocabulary (Medical Subject Headings=MeSH and Excerpta Medicatree=Emtree) and reviewing primary search results. The search strategy is reported in supplementary table 1.

**Study selection**

Search results were imported to EndNote X9 for identification and removal of duplicates. Two researchers independently screened the records in a double-blind mode on Rayyan.ai. Conflicts were resolved through discussion with a third investigator. The same two researchers initially analyzed the title, then the abstract, and, in case of doubts, the full report of each retrieved article, documenting the reasons for the exclusion of reports. Multiple reports of the same study were collated under the same study name. Reference lists of identified studies and previously published meta-analyses were evaluated to identify any further relevant articles. Selected reports/trials were validated by 2 independent reviewers and a third for conflicts. A standardized pre-piloted data extraction form was used to extract the trial data (supplementary figure 1).

**Estimation of hazard ratio and its standard error from relative risk reductions**

An estimate of the hazard ratio and corresponding 95% confidence interval was obtained under the assumptions of an Exponential (or Poisson) survival model using the published length of the follow-up, total events at the end of follow-up, and total number of individuals in the two arms of the trial.

In any published trial, if not presented, the events and sample size were used to derive the event probability (or its complement survival) at the end of the study period. Subsequently, the event rate or hazard from the published survival probability was derived in each arm of the trial.

Following established methods in the survival analysis literature, the analytical framework described below was applied.

In an exponential survival model, the survival probability decreases over follow-up time $t$ according to the event hazard $\lambda$ as follows $S(t)=e^{-\lambda t}$. Therefore, given a survival probability at one point in time, the hazard can be derived indirectly as $\lambda=-\frac{\ln\left( S\left( t \right) \right)}{t}$. In a simulation study, it can be shown that if the censoring mechanism is approximately the same, the derived hazard ratio is unbiased.

|  | **Control** | **Intervention** |
| --- | --- | --- |
| Sample size | $n_{0}$ | $n_{1}$ |
| Events | $c_{0}$ | $c_{1}$ |
| Follow-up time | $t_{0}$ | $t_{1}$ |
| Event probability | $c_{0}/n_{0}$ | $c_{1}/n_{1}$ |
| Survival probability | $S\left( t_{0} \right)=1-c_{0}/n_{0}$ | $S\left( t_{1} \right)=1-c_{1}/n_{1}$ |
| Event Rate | $\lambda_{0}=-\frac{\ln\left( S\left( t_{0} \right) \right)}{t_{0}}$ | $\lambda_{1}=-\frac{\ln\left( S\left( t_{1} \right) \right)}{t_{1}}$ |

A maximum likelihood estimate of the hazard ratio, on the natural log scale, quantifying the treatment effect is obtained as ratio of event rates

$$\ln\left( \mathrm{HR} \right)= ln\left( \frac{\lambda_{1}}{\lambda_{0}} \right)$$

and an estimate of its standard error, inversely related to the events in both groups, is obtained as follows

$$SE(ln \left( \mathrm{HR} \right))= \sqrt{\frac{1}{c_{1}}+\frac{1}{c_{0}}}$$

A 95% confidence interval for the hazard ratio can be obtained by exponentiating the 0.025 and 0.975 quantiles of confidence for the ln hazard ratio

$$e^{\ln\left( \frac{\lambda_{1}}{\lambda_{0}} \right)\pm1.96\sqrt{\frac{1}{c_{1}}+\frac{1}{c_{0}}}}$$

To fully explain the approach followed in our analysis, an example from the PROGRESS trial with all-cause mortality as endpoint is provided below.

During 4 years of follow-up, among 3051 patients assigned to perindopril (ACE-I) 306 died whereas among 3054 patients assigned to the placebo 319 died.

|  | **Control** | **Intervention** |
| --- | --- | --- |
| Sample size | $3054$ | $3051$ |
| Events | $319$ | $306$ |
| Follow-up time | $4$ years | $4$ years |
| Event probability | $319/3054$ | $306/3051$ |
| Survival probability | $S\left( 4 \right)=1-\frac{319}{3054}=$0.8955 | $S\left( 4 \right)=1-\frac{306}{3051}=0.8997$ |
| Event Rate | $\lambda_{0}=-\frac{\ln\left( 0.8955 \right)}{4}=0.0276$ | $\lambda_{1}=-\frac{\ln\left( 0.8997 \right)}{4}=0.0264$ |

The contribution of this PROGRESS trial to the meta-analysis is quantified by the following ln hazard ratio and its standard error

$$\ln\left( \mathrm{HR} \right)= ln\left( \frac{0.0264}{0.0276} \right)=-0.0429$$

$$SE(ln \left( \mathrm{HR} \right))= \sqrt{\frac{1}{306}+\frac{1}{319}}=0.080$$

To evaluate and compare with other studies in a forest plot, we exponentiate and derive an estimate of the hazard ratio and its 95% confidence interval

$$HR= e^{-0.0429}=0.9580$$

$$95\% CI for the HR= e^{-0.0429\pm1.96(0.080)}=0.8190, 1.1207$$

In summary, using this approach the treatment effect was expressed in a consistent way across studies, independently of what has been published (i.e. counts, relative risk reduction). To provide ful transparency on this analytical approach, a user-friendly package “ctohri”, implementing the calculations above, has been shared on SSC archive for Stata users.

**Statistical analyses**

The summary event rates for cardiovascular mortality, all-cause mortality and MACEs in the placebo group were compared between ACE-Is and ARB trials to assess if patients included in ACE-I or ARB trials carried a similar risk for these events at the time of study recruitment.

To address the primary objective of our meta-analysis, we initially used random-effects models to separately estimate the summary hazard ratios (HRs) with 95% confidence intervals (CIs) for ACEI and ARB trials, with parameter estimates obtained via restricted maximum likelihood. Subsequently, the hypothesis of a homogeneous treatment effect between ACEI and ARB (secondary objective) was tested for each outcome with a Cochran’s Q statistic with a *p*-value derived from a Chi-Square distribution with 1 degree of freedom. The same statistical test was applied to evaluate differences in the rates of cardiovascular mortality, all-cause mortality, and major adverse cardiovascular events (MACE) between the placebo groups of ACE inhibitor and ARB trials. Heterogeneity in treatment effect within the trials testing ACEI and those on ARB was separately examined with I^2^ statistics. I^2^ ranging between 50% and 90% was interpreted as representing substantial heterogeneity, whereas an I^2^ ranging between 75% and 100% represented considerable heterogeneity. Forest plots were used to display the results.

To assess the robustness of the main results, a leave-one-out meta-analysis was conducted by iteratively repeating the meta-analysis while excluding one study at a time. All statistical analysis and graphs were produced using Stata (StataCorp. 2023. *Stata Statistical Software: Release 18*. College Station, TX: StataCorp LLC).

**SUPPLEMENTARY TABLES**

**Supplementary Table 1.** Search strategy adopted in the different database

**BOX 1. Search strategy for CENTRAL**

Advanced Search/Search manager

#1 [mh Hypertension] or [mh "Coronary Artery Disease"] or [mh "Myocardial Infarction"] or [mh "Heart Failure"] or [mh Stroke] or [mh "Ischemic Attack, Transient"] or [mh "Brain Ischemia"] or [mh "Brain Infarction"] or [mh "Brain Stem Infarctions"] or [mh "Cerebral Infarction"] or [mh "Diabetes Mellitus, Type 2"] or [mh "Renal Insufficiency, Chronic"] or (High Blood Pressure* or Hypertensi* or Coronary Artery Disease* or CAD or Left Main Disease* or Left Main Coronary Disease* or Coronary Arteriosclero* or Coronary Atherosclero* or Myocardial Infarct* or MI or MINOCA or NSTEMI or STEMI or Cardiogenic Shock* or Heart Attack* or Stroke* or Heart Failure* or Cardiac Failure* or Heart Decompensation* or Myocardial Failure* or Cerebrovascular Accident* or CVA or CVAs or Brain Vascular Accident* or Apoplexy or Brain Infarct* or Brain Stem Infarct* or Cerebral Infarct* or TIA or TIAs or Transient Ischemic Attack* or Transient Ischaemic Attack* or Transient Brainstem Ischemia* or Transient Brainstem Ischaemia* or Transient Cerebral Ischemia* or Transient Cerebral Ischaemia* or Brain Ischemia* or Brain Ischaemia* or Anterior Cerebral Circulation Infarct* or Brain Stem Infarct* or Brainstem Infarct* or Brain Venous Infarct* or Ischaemic Encephalopath* or Ischemic Encephalopath* or Subcortical Infarct* or Choroidal Artery Infarct* or Diabet* or NIDDM or T2DM or Chronic Renal Insufficienc* or Chronic Kidney Insufficienc* or Chronic Kidney Disease* or CKD or Chronic Renal Disease* or Chronic Kidney Failure* or Chronic Renal Failure* or Nephropath*):ti,ab

#2 [mh "Angiotensin-Converting Enzyme Inhibitors"] or [mh "Vasopeptidase Inhibitors"] or [mh Captopril] or [mh Cilazapril] or [mh Enalapril] or [mh Enalaprilat] or [mh Fosinopril] or [mh Lisinopril] or [mh Perindopril] or [mh Quinapril] or [mh Ramipril] or [mh Teprotide] or (Angiotensin-Converting Enzyme Antagonist* or Angiotensin-Converting Enzyme Block* or Angiotensin-Converting Enzyme Inhibit* or Kininase II Antagonist* or Kininase II Block* or Kininase II Inhibit* or Angiotensin I-Converting Enzyme Antagonist* or Angiotensin I-Converting Enzyme Block* or Angiotensin I-Converting Enzyme Inhibit* or ACE Antagonist* or ACE Block* or ACE Inhibit* or ACEI or ACEIs or ACE-NEP Antagonist* or ACE-NEP Block* or ACE-NEP Inhibit* or Vasopeptidase Antagonist* or Vasopeptidase Block* or Vasopeptidase Inhibit* or Dipeptidyl Carboxypeptidase Antagonist* or Dipeptidyl Carboxypeptidase Block* or Dipeptidyl Carboxypeptidase Inhibit* or Dipeptidyl Carboxypeptidase I Antagonist* or Dipeptidyl Carboxypeptidase I Block* or Dipeptidyl Carboxypeptidase I Inhibit* or Peptidyl Dipeptidase Antagonist* or Peptidyl Dipeptidase Block* or Peptidyl Dipeptidase Inhibit* or Peptidyldipeptide Hydrolase Antagonist* or Peptidyldipeptide Hydrolase Block* or Peptidyldipeptide Hydrolase Inhibit* or "Pres IV" or "Tenso Stop" or "VPP Peptide" or Accupril* or Aceon or Acovil* or Alacepril* or Altace or Altiopril* or Ancovenin or Aracepril* or Benazapril* or Benazepril* or Benezapril* or Benzazepril* or Berlipril* or Briem or Capoten or Captopril* or Carasel or Ceranapril* or Ceronapril* or Cibacen* or Cilazapril* or Coversyl or Deacetylalacepril* or Delapril* or Delix or Derapril* or Dynacil* or Enalapril* or Enap or Epicaptopril* or Fasidotril* or Fempress or Foroxymithine or Fosenopril* or Fosfenopril* or Fosinil* or Fosinopril* or Fosinorm or Fositen* or Fozitec or Gemopatril* or Gopten or Hiperlex or Idapril* or Idrapril* or Ilepatril* or Imidapril* or Indolapril* or Inhibace or Labopal or Libenzapril* or Lisidigal or Lisinopril* or Listril* or Lopirin or Lopril* or Lotensin or Lysinopril* or Mavik or Mixanpril* or Moex or Moexipril* or Monopril* or Moveltipril* or Newace or Nitrosocaptopril* or Novatec or Odrik or Omapatril* or Pentopril* or Perdix or Perindo* or Perstarium or Pirindopril* or Pivopril* or Prilace or Prinivil* or Quadropril* or Quinapril* or Ramace or Ramipril* or Ramitac or Ramiwin or Renitec or Renitek or Renormax or Renpress or Rentiapril* or Sampatril* or Spirapril* or Staril* or Tanatril* or Temocapril* or Tensocardil* or Teprotide or Trandolapril* or Triatec or Tritace or Udrik or Univasc or Utibapril* or Valine-Proline-Proline or Val-Pro-Pro or Valyl-Prolyl-Proline or Vanlev or Vasotec or Vesdil* or Xanef or Zabicipril* or Zabien or Zestril* or Zofenil* or Zofenopril* or Zofil*):ti,ab

#3 [mh "Angiotensin Receptor Antagonists"] or [mh "Angiotensin II Type 2 Receptor Blockers"] or [mh "Angiotensin II Type 1 Receptor Blockers"] or [mh "Amlodipine Besylate, Olmesartan Medoxomil Drug Combination"] or [mh Irbesartan] or [mh Losartan] or [mh "Olmesartan Medoxomil"] or [mh Saralasin] or [mh Telmisartan] or [mh Valsartan] or (Angiotensin Receptor Antagonist* or Angiotensin Receptor Block* or Angiotensin Receptor Inhibit* or Angiotensin II Receptor Antagonist* or Angiotensin II Receptor Block* or Angiotensin II Receptor Inhibit* or Angiotensin 2 Receptor Antagonist* or Angiotensin 2 Receptor Block* or Angiotensin 2 Receptor Inhibit* or Angiotensin 2 Type 1 Receptor Antagonist* or Angiotensin 2 Type 1 Receptor Block* or Angiotensin 2 Type 1 Receptor Inhibit* or Angiotensin II Type 1 Receptor Antagonist* or Angiotensin II Type 1 Receptor Block* or Angiotensin II Type 1 Receptor Inhibit* or Angiotensin 2 Type 2 Receptor Antagonist* or Angiotensin 2 Type 2 Receptor Block* or Angiotensin 2 Type 2 Receptor Inhibit* or Angiotensin II Type 2 Receptor Antagonist* or Angiotensin II Type 2 Receptor Block* or Angiotensin II Type 2 Receptor Inhibit* or Abitesartan or Allisartan or Aprovel or Atacand or Avapro or Azilsartan or Azor or Benicar or Candesartan or Cardosten or Cozaar or Diovan or Edarbi or Elisartan or Embusartan or Enoltasosartan or Eprosartan or Fimasartan or Fonsartan or Forasartan or Irbesartan or Kalpress or Karvea or Losartan or Micardis or Milfasartan or Miten or Nisis or Olmesartan or Olmetec or Olodanrigan or Omesartan or Pomisartan or Pratosartan or Pritor or Provas or Ripisartan or Saprisartan or Saralasin or Sartan* or Sparsentan or Tareg or Tasosartan or Telmisartan or Teveten or Tonlamarsen or Vals or Valsartan or Votum or Zolasartan):ti,ab

#4 #2 OR #3

#5 [mh "Double-Blind Method"] or (Double-Blind* or Doubleblind* or Double-Mask* or Double-Dum* or Doubledum* or Triple-Blind* or Trippleblind* or Treble-Blind* or Trebleblind* or Quadruple-Blind* or Quadrupleblind* or Blind* or Mask* or Dumm*):ti,ab

#6 [mh Mortality] or [mh "Hospital Mortality"] or [mh Death] or [mh "Cause of Death"] or [mh "Fatal Outcome"] or [mh "Survival Rate"] or [mh "Death, Sudden"] or [mh "Death, Sudden, Cardiac"] or [mh /MO] or (Mortalit* or "End Of Life" or Death* or Fatal* or Survival* OR Sudden Cardiac Arrest* or Died or Dead):ti,ab

#7 #1 AND #4 AND #5 AND #6 in Trials

**BOX 2. Search strategy for ClinicalTrials.gov**

Advanced Search

**Search #1:**

***Condition or disease^*^:*** High Blood Pressure OR Coronary Artery Disease OR Heart Infarction OR Heart Failure OR Stroke OR Transient Ischemic Attack OR Brain Ischemia OR Brain Infarction OR Diabetes OR Heart Attack OR Chronic Kidney OR Chronic Renal OR Nephropathy

***Study type:*** Interventional Studies (Clinical Trials)

***Other terms:*** Randomized OR Randomized OR RCT

***Intervention/treatment^*^:*** Azilsartan OR Benazepril OR Candesartan OR Captopril OR Delapril OR Enalapril OR Eprosartan OR Fimasartan OR Fosinopril OR Fosinopril OR Imidapril OR Irbesartan OR Lisinopril OR Losartan OR Olmesartan OR Perindopril OR Quinapril OR Ramipril

**Search #2:**

**Condition or disease^*^:** High Blood Pressure OR Coronary Artery Disease OR Heart Infarction OR Heart Failure OR Stroke OR Transient Ischemic Attack OR Brain Ischemia OR Brain Infarction OR Diabetes OR Heart Attack OR Chronic Kidney OR Chronic Renal OR Nephropathy

**Study type:** Interventional Studies (Clinical Trials)

**Intervention/treatment^*^:** Telmisartan OR Trandolapril OR Valsartan

**BOX 3. Search strategy for Embase**

Database: Embase <1974 to date of search>

1 Hypertension/ or Coronary Artery Disease/ or Heart Infarction/ or Heart Failure/ or Cerebrovascular Accident/ or Transient Ischemic Attack/ or Brain Ischemia/ or Brain Infarction/ or Brain Stem Infarction/ or Diabetes Mellitus/ or Non Insulin Dependent Diabetes Mellitus/ or Chronic Kidney Failure/ or (High Blood Pressure* or Hypertensi* or Coronary Artery Disease* or CAD or Left Main Disease* or Left Main Coronary Disease* or Coronary Arteriosclero* or Coronary Atherosclero* or Myocardial Infarct* or MI or MINOCA or NSTEMI or STEMI or Cardiogenic Shock* or Heart Attack* or Stroke* or Heart Failure* or Cardiac Failure* or Heart Decompensation* or Myocardial Failure* or Cerebrovascular Accident* or CVA or CVAs or Brain Vascular Accident* or Apoplexy or Brain Infarct* or Brain Stem Infarct* or Cerebral Infarct* or TIA or TIAs or Transient Ischemic Attack* or Transient Ischaemic Attack* or Transient Brainstem Ischemia* or Transient Brainstem Ischaemia* or Transient Cerebral Ischemia* or Transient Cerebral Ischaemia* or Brain Ischemia* or Brain Ischaemia* or Anterior Cerebral Circulation Infarct* or Brain Stem Infarct* or Brainstem Infarct* or Brain Venous Infarct* or Ischaemic Encephalopath* or Ischemic Encephalopath* or Subcortical Infarct* or Choroidal Artery Infarct* or Diabet* or NIDDM or T2DM or Chronic Renal Insufficienc* or Chronic Kidney Insufficienc* or Chronic Kidney Disease* or CKD or Chronic Renal Disease* or Chronic Kidney Failure* or Chronic Renal Failure* or Nephropath*).ti,ab.

2 exp Dipeptidyl Carboxypeptidase Inhibitor/ or Vasopeptidase Inhibitor/ or Benazepril/ or Captopril/ or Cilazapril/ or Delapril/ or exp Enalapril/ or Enalaprilat/ or Fosinopril/ or Lisinopril/ or Moexipril/ or Pentopril/ or Perindopril/ or Pivopril/ or Quinapril/ or Ramipril/ or Temocapril/ or Teprotide/ or Trandolapril/ or Zabicipril/ or Zofenopril/ or Alacepril/ or Altiopril/ or Ancovenin/ or Benazeprilat/ or Ceranapril/ or Cilazaprilat/ or Deacetylalacepril/ or Epicaptopril/ or Fasidotril/ or Fasidotrilat/ or Foroxymithine/ or Fosinoprilat/ or Gemopatrilat/ or Idrapril/ or Ilepatril/ or Imidapril/ or Imidaprilat/ or Indolapril/ or Libenzapril/ or Moexiprilat/ or Omapatrilat/ or Pentoprilat/ or Perindoprilat/ or Quinaprilat/ or Ramiprilat/ or Rentiapril/ or "S Nitrosocaptopril"/ or Sampatrilat/ or Spirapril/ or Spiraprilat/ or Temocaprilat/ or Trandolaprilat/ or Utibapril/ or Utibaprilat/ or Zabiciprilat/ or Zofenoprilat/ or (Angiotensin-Converting Enzyme Antagonist* or Angiotensin-Converting Enzyme Block* or Angiotensin-Converting Enzyme Inhibit* or Kininase II Antagonist* or Kininase II Block* or Kininase II Inhibit* or Angiotensin I-Converting Enzyme Antagonist* or Angiotensin I-Converting Enzyme Block* or Angiotensin I-Converting Enzyme Inhibit* or ACE Antagonist* or ACE Block* or ACE Inhibit* or ACEI or ACEIs or ACE-NEP Antagonist* or ACE-NEP Block* or ACE-NEP Inhibit* or Vasopeptidase Antagonist* or Vasopeptidase Block* or Vasopeptidase Inhibit* or Dipeptidyl Carboxypeptidase Antagonist* or Dipeptidyl Carboxypeptidase Block* or Dipeptidyl Carboxypeptidase Inhibit* or Dipeptidyl Carboxypeptidase I Antagonist* or Dipeptidyl Carboxypeptidase I Block* or Dipeptidyl Carboxypeptidase I Inhibit* or Peptidyl Dipeptidase Antagonist* or Peptidyl Dipeptidase Block* or Peptidyl Dipeptidase Inhibit* or Peptidyldipeptide Hydrolase Antagonist* or Peptidyldipeptide Hydrolase Block* or Peptidyldipeptide Hydrolase Inhibit* or "Pres IV" or "Tenso Stop" or "VPP Peptide" or Accupril* or Aceon or Acovil* or Alacepril* or Altace or Altiopril* or Ancovenin or Aracepril* or Benazapril* or Benazepril* or Benezapril* or Benzazepril* or Berlipril* or Briem or Capoten or Captopril* or Carasel or Ceranapril* or Ceronapril* or Cibacen* or Cilazapril* or Coversyl or Deacetylalacepril* or Delapril* or Delix or Derapril* or Dynacil* or Enalapril* or Enap or Epicaptopril* or Fasidotril* or Fempress or Foroxymithine or Fosenopril* or Fosfenopril* or Fosinil* or Fosinopril* or Fosinorm or Fositen* or Fozitec or Gemopatril* or Gopten or Hiperlex or Idapril* or Idrapril* or Ilepatril* or Imidapril* or Indolapril* or Inhibace or Labopal or Libenzapril* or Lisidigal or Lisinopril* or Listril* or Lopirin or Lopril* or Lotensin or Lysinopril* or Mavik or Mixanpril* or Moex or Moexipril* or Monopril* or Moveltipril* or Newace or Nitrosocaptopril* or Novatec or Odrik or Omapatril* or Pentopril* or Perdix or Perindo* or Perstarium or Pirindopril* or Pivopril* or Prilace or Prinivil* or Quadropril* or Quinapril* or Ramace or Ramipril* or Ramitac or Ramiwin or Renitec or Renitek or Renormax or Renpress or Rentiapril* or Sampatril* or Spirapril* or Staril* or Tanatril* or Temocapril* or Tensocardil* or Teprotide or Trandolapril* or Triatec or Tritace or Udrik or Univasc or Utibapril* or Valine-Proline-Proline or Val-Pro-Pro or Valyl-Prolyl-Proline or Vanlev or Vasotec or Vesdil* or Xanef or Zabicipril* or Zabien or Zestril* or Zofenil* or Zofenopril* or Zofil*).ti,ab.

3 exp Angiotensin Receptor Antagonist/ or Angiotensin 2 Receptor Antagonist/ or Angiotensin 1 Receptor Antagonist/ or "Amlodipine Plus Olmesartan"/ or Irbesartan/ or Losartan/ or Olmesartan/ or Saralasin/ or Telmisartan/ or Valsartan/ or Abitesartan/ or Azilsartan/ or Candesartan/ or Elisartan/ or Embusartan/ or Eprosartan/ or Fimasartan/ or Fonsartan/ or Forasartan/ or Milfasartan/ or Olodanrigan/ or Pomisartan/ or Pratosartan/ or Ripisartan/ or Saprisartan/ or Sparsentan/ or Tasosartan/ or Tonlamarsen/ or Zolasartan/ or (Angiotensin Receptor Antagonist* or Angiotensin Receptor Block* or Angiotensin Receptor Inhibit* or Angiotensin II Receptor Antagonist* or Angiotensin II Receptor Block* or Angiotensin II Receptor Inhibit* or Angiotensin 2 Receptor Antagonist* or Angiotensin 2 Receptor Block* or Angiotensin 2 Receptor Inhibit* or Angiotensin 2 Type 1 Receptor Antagonist* or Angiotensin 2 Type 1 Receptor Block* or Angiotensin 2 Type 1 Receptor Inhibit* or Angiotensin II Type 1 Receptor Antagonist* or Angiotensin II Type 1 Receptor Block* or Angiotensin II Type 1 Receptor Inhibit* or Angiotensin 2 Type 2 Receptor Antagonist* or Angiotensin 2 Type 2 Receptor Block* or Angiotensin 2 Type 2 Receptor Inhibit* or Angiotensin II Type 2 Receptor Antagonist* or Angiotensin II Type 2 Receptor Block* or Angiotensin II Type 2 Receptor Inhibit* or Abitesartan or Allisartan or Aprovel or Atacand or Avapro or Azilsartan or Azor or Benicar or Candesartan or Cardosten or Cozaar or Diovan or Edarbi or Elisartan or Embusartan or Enoltasosartan or Eprosartan or Fimasartan or Fonsartan or Forasartan or Irbesartan or Kalpress or Karvea or Losartan or Micardis or Milfasartan or Miten or Nisis or Olmesartan or Olmetec or Olodanrigan or Omesartan or Pomisartan or Pratosartan or Pritor or Provas or Ripisartan or Saprisartan or Saralasin or Sartan* or Sparsentan or Tareg or Tasosartan or Telmisartan or Teveten or Tonlamarsen or Vals or Valsartan or Votum or Zolasartan).ti,ab.

4 2 or 3

5 Randomized controlled trial/ or Controlled clinical study/ or randomization/ or intermethod comparison/ or double blind procedure/ or human experiment/ or (random$ or placebo or (open adj label) or ((double or single or doubly or singly) adj (blind or blinded or blindly)) or parallel group$1 or crossover or cross over or ((assign$ or match or matched or allocation) adj5 (alternate or group$1 or intervention$1 or patient$1 or subject$1 or participant$1)) or assigned or allocated or (controlled adj7 (study or design or trial)) or volunteer or volunteers).ti,ab. or (compare or compared or comparison or trial).ti. or ((evaluated or evaluate or evaluating or assessed or assess) and (compare or compared or comparing or comparison)).ab.

6 (random$ adj sampl$ adj7 ("cross section$" or questionnaire$1 or survey$ or database$1)).ti,ab. not (comparative study/ or controlled study/ or randomi?ed controlled.ti,ab. or randomly assigned.ti,ab.)

7 Cross-sectional study/ not (randomized controlled trial/ or controlled clinical study/ or controlled study/ or (randomi?ed controlled or control group$1).ti,ab.)

8 (((case adj control$) and random$) not randomi?ed controlled).ti,ab.

9 (Systematic review not (trial or study)).ti.

10 (nonrandom$ not random$).ti,ab.

11 ("Random field$" or (random cluster adj3 sampl$)).ti,ab.

12 (review.ab. and review.pt.) not trial.ti.

13 "we searched".ab. and (review.ti. or review.pt.)

14 ("update review" or (databases adj4 searched)).ab.

15 (rat or rats or mouse or mice or swine or porcine or murine or sheep or lambs or pigs or piglets or rabbit or rabbits or cat or cats or dog or dogs or cattle or bovine or monkey or monkeys or trout or marmoset$1).ti. and animal experiment/

16 Animal experiment/ not (human experiment/ or human/)

17 or/6-16

18 5 not 17

19 Double Blind Procedure/ or Triple Blind Procedure/ or (Double-Blind* or Doubleblind* or Double-Mask* or Double-Dum* or Doubledum* or Triple-Blind* or Trippleblind* or Treble-Blind* or Trebleblind* or Quadruple-Blind* or Quadrupleblind* or Blind* or Mask* or Dumm*).ti,ab.

20 exp Mortality/ or exp Hospital Mortality/ or Cardiovascular Mortality/ or exp Mortality Rate/ or exp Death/ or "Cause of Death"/ or Fatality/ or Survival Rate/ or Sudden Death/ or Sudden Cardiac Death/ or (Mortalit* or "End Of Life" or Death* or Fatal* or Survival* or Sudden Cardiac Arrest* or Died or Dead).ti,ab.

21 1 and 4 and 18 and 19 and 20

22 limit 21 to Embase (2168)

**BOX 4. Search strategy for MEDLINE**

Database: Ovid MEDLINE(R) ALL <1946 to date of search >

1 Hypertension/ or Coronary Artery Disease/ or Myocardial Infarction/ or Heart Failure/ or Stroke/ or Ischemic Attack, Transient/ or Brain Ischemia/ or Brain Infarction/ or Brain Stem Infarctions/ or Cerebral Infarction/ or Diabetes Mellitus, Type 2/ or Renal Insufficiency, Chronic/ or (High Blood Pressure* or Hypertensi* or Coronary Artery Disease* or CAD or Left Main Disease* or Left Main Coronary Disease* or Coronary Arteriosclero* or Coronary Atherosclero* or Myocardial Infarct* or MI or MINOCA or NSTEMI or STEMI or Cardiogenic Shock* or Heart Attack* or Stroke* or Heart Failure* or Cardiac Failure* or Heart Decompensation* or Myocardial Failure* or Cerebrovascular Accident* or CVA or CVAs or Brain Vascular Accident* or Apoplexy or Brain Infarct* or Brain Stem Infarct* or Cerebral Infarct* or TIA or TIAs or Transient Ischemic Attack* or Transient Ischaemic Attack* or Transient Brainstem Ischemia* or Transient Brainstem Ischaemia* or Transient Cerebral Ischemia* or Transient Cerebral Ischaemia* or Brain Ischemia* or Brain Ischaemia* or Anterior Cerebral Circulation Infarct* or Brain Stem Infarct* or Brainstem Infarct* or Brain Venous Infarct* or Ischaemic Encephalopath* or Ischemic Encephalopath* or Subcortical Infarct* or Choroidal Artery Infarct* or Diabet* or NIDDM or T2DM or Chronic Renal Insufficienc* or Chronic Kidney Insufficienc* or Chronic Kidney Disease* or CKD or Chronic Renal Disease* or Chronic Kidney Failure* or Chronic Renal Failure* or Nephropath*).ti,ab.

2 exp Angiotensin-Converting Enzyme Inhibitors/ or Vasopeptidase Inhibitors/ or Captopril/ or Cilazapril/ or exp Enalapril/ or Enalaprilat/ or Fosinopril/ or Lisinopril/ or Perindopril/ or Quinapril/ or Ramipril/ or Teprotide/ or (Angiotensin-Converting Enzyme Antagonist* or Angiotensin-Converting Enzyme Block* or Angiotensin-Converting Enzyme Inhibit* or Kininase II Antagonist* or Kininase II Block* or Kininase II Inhibit* or Angiotensin I-Converting Enzyme Antagonist* or Angiotensin I-Converting Enzyme Block* or Angiotensin I-Converting Enzyme Inhibit* or ACE Antagonist* or ACE Block* or ACE Inhibit* or ACEI or ACEIs or ACE-NEP Antagonist* or ACE-NEP Block* or ACE-NEP Inhibit* or Vasopeptidase Antagonist* or Vasopeptidase Block* or Vasopeptidase Inhibit* or Dipeptidyl Carboxypeptidase Antagonist* or Dipeptidyl Carboxypeptidase Block* or Dipeptidyl Carboxypeptidase Inhibit* or Dipeptidyl Carboxypeptidase I Antagonist* or Dipeptidyl Carboxypeptidase I Block* or Dipeptidyl Carboxypeptidase I Inhibit* or Peptidyl Dipeptidase Antagonist* or Peptidyl Dipeptidase Block* or Peptidyl Dipeptidase Inhibit* or Peptidyldipeptide Hydrolase Antagonist* or Peptidyldipeptide Hydrolase Block* or Peptidyldipeptide Hydrolase Inhibit* or "Pres IV" or "Tenso Stop" or "VPP Peptide" or Accupril* or Aceon or Acovil* or Alacepril* or Altace or Altiopril* or Ancovenin or Aracepril* or Benazapril* or Benazepril* or Benezapril* or Benzazepril* or Berlipril* or Briem or Capoten or Captopril* or Carasel or Ceranapril* or Ceronapril* or Cibacen* or Cilazapril* or Coversyl or Deacetylalacepril* or Delapril* or Delix or Derapril* or Dynacil* or Enalapril* or Enap or Epicaptopril* or Fasidotril* or Fempress or Foroxymithine or Fosenopril* or Fosfenopril* or Fosinil* or Fosinopril* or Fosinorm or Fositen* or Fozitec or Gemopatril* or Gopten or Hiperlex or Idapril* or Idrapril* or Ilepatril* or Imidapril* or Indolapril* or Inhibace or Labopal or Libenzapril* or Lisidigal or Lisinopril* or Listril* or Lopirin or Lopril* or Lotensin or Lysinopril* or Mavik or Mixanpril* or Moex or Moexipril* or Monopril* or Moveltipril* or Newace or Nitrosocaptopril* or Novatec or Odrik or Omapatril* or Pentopril* or Perdix or Perindo* or Perstarium or Pirindopril* or Pivopril* or Prilace or Prinivil* or Quadropril* or Quinapril* or Ramace or Ramipril* or Ramitac or Ramiwin or Renitec or Renitek or Renormax or Renpress or Rentiapril* or Sampatril* or Spirapril* or Staril* or Tanatril* or Temocapril* or Tensocardil* or Teprotide or Trandolapril* or Triatec or Tritace or Udrik or Univasc or Utibapril* or Valine-Proline-Proline or Val-Pro-Pro or Valyl-Prolyl-Proline or Vanlev or Vasotec or Vesdil* or Xanef or Zabicipril* or Zabien or Zestril* or Zofenil* or Zofenopril* or Zofil*).ti,ab.

3 exp Angiotensin Receptor Antagonists/ or Angiotensin II Type 2 Receptor Blockers/ or Angiotensin II Type 1 Receptor Blockers/ or "Amlodipine Besylate, Olmesartan Medoxomil Drug Combination"/ or Irbesartan/ or Losartan/ or Olmesartan Medoxomil/ or Saralasin/ or Telmisartan/ or Valsartan/ or (Angiotensin Receptor Antagonist* or Angiotensin Receptor Block* or Angiotensin Receptor Inhibit* or Angiotensin II Receptor Antagonist* or Angiotensin II Receptor Block* or Angiotensin II Receptor Inhibit* or Angiotensin 2 Receptor Antagonist* or Angiotensin 2 Receptor Block* or Angiotensin 2 Receptor Inhibit* or Angiotensin 2 Type 1 Receptor Antagonist* or Angiotensin 2 Type 1 Receptor Block* or Angiotensin 2 Type 1 Receptor Inhibit* or Angiotensin II Type 1 Receptor Antagonist* or Angiotensin II Type 1 Receptor Block* or Angiotensin II Type 1 Receptor Inhibit* or Angiotensin 2 Type 2 Receptor Antagonist* or Angiotensin 2 Type 2 Receptor Block* or Angiotensin 2 Type 2 Receptor Inhibit* or Angiotensin II Type 2 Receptor Antagonist* or Angiotensin II Type 2 Receptor Block* or Angiotensin II Type 2 Receptor Inhibit* or Abitesartan or Allisartan or Aprovel or Atacand or Avapro or Azilsartan or Azor or Benicar or Candesartan or Cardosten or Cozaar or Diovan or Edarbi or Elisartan or Embusartan or Enoltasosartan or Eprosartan or Fimasartan or Fonsartan or Forasartan or Irbesartan or Kalpress or Karvea or Losartan or Micardis or Milfasartan or Miten or Nisis or Olmesartan or Olmetec or Olodanrigan or Omesartan or Pomisartan or Pratosartan or Pritor or Provas or Ripisartan or Saprisartan or Saralasin or Sartan* or Sparsentan or Tareg or Tasosartan or Telmisartan or Teveten or Tonlamarsen or Vals or Valsartan or Votum or Zolasartan).ti,ab.

4 2 or 3

5 ((Randomized Controlled Trial or Controlled Clinical Trial).pt. or (Randomi?ed or Placebo or Randomly or Trial or Groups).ab.) not (exp Animals/ not Humans.sh.)

6 Double-Blind Method/ or (Double-Blind* or Doubleblind* or Double-Mask* or Double-Dum* or Doubledum* or Triple-Blind* or Trippleblind* or Treble-Blind* or Trebleblind* or Quadruple-Blind* or Quadrupleblind* or Blind* or Mask* or Dumm*).ti,ab.

7 exp Mortality/ or Hospital Mortality/ or Death/ or "Cause of Death"/ or Fatal Outcome/ or Survival Rate/ or Death, Sudden/ or Death, Sudden, Cardiac/ or Mortality.fs. or (Mortalit* or "End Of Life" or Death* or Fatal* or Survival* or Sudden Cardiac Arrest* or Died or Dead).ti,ab.

8 and/1,4-7

**BOX 5. Search strategy for Science Citation Index-Expanded**

Database: Web of Science Core Collection < WOS.SCI 1900 to 2023 >

1: High Blood Pressure* or Hypertensi* or Coronary Artery Disease* or CAD or Left Main Disease* or Left Main Coronary Disease* or Coronary Arteriosclero* or Coronary Atherosclero* or Myocardial Infarct* or MI or MINOCA or NSTEMI or STEMI or Cardiogenic Shock* or Heart Attack* or Stroke* or Heart Failure* or Cardiac Failure* or Heart Decompensation* or Myocardial Failure* or Cerebrovascular Accident* or CVA or CVAs or Brain Vascular Accident* or Apoplexy or Brain Infarct* or Brain Stem Infarct* or Cerebral Infarct* or TIA or TIAs or Transient Ischemic Attack* or Transient Ischaemic Attack* or Transient Brainstem Ischemia* or Transient Brainstem Ischaemia* or Transient Cerebral Ischemia* or Transient Cerebral Ischaemia* or Brain Ischemia* or Brain Ischaemia* or Anterior Cerebral Circulation Infarct* or Brain Stem Infarct* or Brainstem Infarct* or Brain Venous Infarct* or Ischaemic Encephalopath* or Ischemic Encephalopath* or Subcortical Infarct* or Choroidal Artery Infarct* or Diabet* or NIDDM or T2DM or Chronic Renal Insufficienc* or Chronic Kidney Insufficienc* or Chronic Kidney Disease* or CKD or Chronic Renal Disease* or Chronic Kidney Failure* or Chronic Renal Failure* or Nephropath* (Title) OR High Blood Pressure* or Hypertensi* or Coronary Artery Disease* or CAD or Left Main Disease* or Left Main Coronary Disease* or Coronary Arteriosclero* or Coronary Atherosclero* or Myocardial Infarct* or MI or MINOCA or NSTEMI or STEMI or Cardiogenic Shock* or Heart Attack* or Stroke* or Heart Failure* or Cardiac Failure* or Heart Decompensation* or Myocardial Failure* or Cerebrovascular Accident* or CVA or CVAs or Brain Vascular Accident* or Apoplexy or Brain Infarct* or Brain Stem Infarct* or Cerebral Infarct* or TIA or TIAs or Transient Ischemic Attack* or Transient Ischaemic Attack* or Transient Brainstem Ischemia* or Transient Brainstem Ischaemia* or Transient Cerebral Ischemia* or Transient Cerebral Ischaemia* or Brain Ischemia* or Brain Ischaemia* or Anterior Cerebral Circulation Infarct* or Brain Stem Infarct* or Brainstem Infarct* or Brain Venous Infarct* or Ischaemic Encephalopath* or Ischemic Encephalopath* or Subcortical Infarct* or Choroidal Artery Infarct* or Diabet* or NIDDM or T2DM or Chronic Renal Insufficienc* or Chronic Kidney Insufficienc* or Chronic Kidney Disease* or CKD or Chronic Renal Disease* or Chronic Kidney Failure* or Chronic Renal Failure* or Nephropath* (Abstract)

2: Angiotensin-Converting Enzyme Antagonist* or Angiotensin-Converting Enzyme Block* or Angiotensin-Converting Enzyme Inhibit* or Kininase II Antagonist* or Kininase II Block* or Kininase II Inhibit* or Angiotensin I-Converting Enzyme Antagonist* or Angiotensin I-Converting Enzyme Block* or Angiotensin I-Converting Enzyme Inhibit* or ACE Antagonist* or ACE Block* or ACE Inhibit* or ACEI or ACEIs or ACE-NEP Antagonist* or ACE-NEP Block* or ACE-NEP Inhibit* or Vasopeptidase Antagonist* or Vasopeptidase Block* or Vasopeptidase Inhibit* or Dipeptidyl Carboxypeptidase Antagonist* or Dipeptidyl Carboxypeptidase Block* or Dipeptidyl Carboxypeptidase Inhibit* or Dipeptidyl Carboxypeptidase I Antagonist* or Dipeptidyl Carboxypeptidase I Block* or Dipeptidyl Carboxypeptidase I Inhibit* or Peptidyl Dipeptidase Antagonist* or Peptidyl Dipeptidase Block* or Peptidyl Dipeptidase Inhibit* or Peptidyldipeptide Hydrolase Antagonist* or Peptidyldipeptide Hydrolase Block* or Peptidyldipeptide Hydrolase Inhibit* or "Pres IV" or "Tenso Stop" or "VPP Peptide" or Accupril* or Aceon or Acovil* or Alacepril* or Altace or Altiopril* or Ancovenin or Aracepril* or Benazapril* or Benazepril* or Benezapril* or Benzazepril* or Berlipril* or Briem or Capoten or Captopril* or Carasel or Ceranapril* or Ceronapril* or Cibacen* or Cilazapril* or Coversyl or Deacetylalacepril* or Delapril* or Delix or Derapril* or Dynacil* or Enalapril* or Enap or Epicaptopril* or Fasidotril* or Fempress or Foroxymithine or Fosenopril* or Fosfenopril* or Fosinil* or Fosinopril* or Fosinorm or Fositen* or Fozitec or Gemopatril* or Gopten or Hiperlex or Idapril* or Idrapril* or Ilepatril* or Imidapril* or Indolapril* or Inhibace or Labopal or Libenzapril* or Lisidigal or Lisinopril* or Listril* or Lopirin or Lopril* or Lotensin or Lysinopril* or Mavik or Mixanpril* or Moex or Moexipril* or Monopril* or Moveltipril* or Newace or Nitrosocaptopril* or Novatec or Odrik or Omapatril* or Pentopril* or Perdix or Perindo* or Perstarium or Pirindopril* or Pivopril* or Prilace or Prinivil* or Quadropril* or Quinapril* or Ramace or Ramipril* or Ramitac or Ramiwin or Renitec or Renitek or Renormax or Renpress or Rentiapril* or Sampatril* or Spirapril* or Staril* or Tanatril* or Temocapril* or Tensocardil* or Teprotide or Trandolapril* or Triatec or Tritace or Udrik or Univasc or Utibapril* or Valine-Proline-Proline or Val-Pro-Pro or Valyl-Prolyl-Proline or Vanlev or Vasotec or Vesdil* or Xanef or Zabicipril* or Zabien or Zestril* or Zofenil* or Zofenopril* or Zofil* or Angiotensin Receptor Antagonist* or Angiotensin Receptor Block* or Angiotensin Receptor Inhibit* or Angiotensin II Receptor Antagonist* or Angiotensin II Receptor Block* or Angiotensin II Receptor Inhibit* or Angiotensin 2 Receptor Antagonist* or Angiotensin 2 Receptor Block* or Angiotensin 2 Receptor Inhibit* or Angiotensin 2 Type 1 Receptor Antagonist* or Angiotensin 2 Type 1 Receptor Block* or Angiotensin 2 Type 1 Receptor Inhibit* or Angiotensin II Type 1 Receptor Antagonist* or Angiotensin II Type 1 Receptor Block* or Angiotensin II Type 1 Receptor Inhibit* or Angiotensin 2 Type 2 Receptor Antagonist* or Angiotensin 2 Type 2 Receptor Block* or Angiotensin 2 Type 2 Receptor Inhibit* or Angiotensin II Type 2 Receptor Antagonist* or Angiotensin II Type 2 Receptor Block* or Angiotensin II Type 2 Receptor Inhibit* or Abitesartan or Allisartan or Aprovel or Atacand or Avapro or Azilsartan or Azor or Benicar or Candesartan or Cardosten or Cozaar or Diovan or Edarbi or Elisartan or Embusartan or Enoltasosartan or Eprosartan or Fimasartan or Fonsartan or Forasartan or Irbesartan or Kalpress or Karvea or Losartan or Micardis or Milfasartan or Miten or Nisis or Olmesartan or Olmetec or Olodanrigan or Omesartan or Pomisartan or Pratosartan or Pritor or Provas or Ripisartan or Saprisartan or Saralasin or Sartan* or Sparsentan or Tareg or Tasosartan or Telmisartan or Teveten or Tonlamarsen or Vals or Valsartan or Votum or Zolasartan (Title) OR Angiotensin-Converting Enzyme Antagonist* or Angiotensin-Converting Enzyme Block* or Angiotensin-Converting Enzyme Inhibit* or Kininase II Antagonist* or Kininase II Block* or Kininase II Inhibit* or Angiotensin I-Converting Enzyme Antagonist* or Angiotensin I-Converting Enzyme Block* or Angiotensin I-Converting Enzyme Inhibit* or ACE Antagonist* or ACE Block* or ACE Inhibit* or ACEI or ACEIs or ACE-NEP Antagonist* or ACE-NEP Block* or ACE-NEP Inhibit* or Vasopeptidase Antagonist* or Vasopeptidase Block* or Vasopeptidase Inhibit* or Dipeptidyl Carboxypeptidase Antagonist* or Dipeptidyl Carboxypeptidase Block* or Dipeptidyl Carboxypeptidase Inhibit* or Dipeptidyl Carboxypeptidase I Antagonist* or Dipeptidyl Carboxypeptidase I Block* or Dipeptidyl Carboxypeptidase I Inhibit* or Peptidyl Dipeptidase Antagonist* or Peptidyl Dipeptidase Block* or Peptidyl Dipeptidase Inhibit* or Peptidyldipeptide Hydrolase Antagonist* or Peptidyldipeptide Hydrolase Block* or Peptidyldipeptide Hydrolase Inhibit* or "Pres IV" or "Tenso Stop" or "VPP Peptide" or Accupril* or Aceon or Acovil* or Alacepril* or Altace or Altiopril* or Ancovenin or Aracepril* or Benazapril* or Benazepril* or Benezapril* or Benzazepril* or Berlipril* or Briem or Capoten or Captopril* or Carasel or Ceranapril* or Ceronapril* or Cibacen* or Cilazapril* or Coversyl or Deacetylalacepril* or Delapril* or Delix or Derapril* or Dynacil* or Enalapril* or Enap or Epicaptopril* or Fasidotril* or Fempress or Foroxymithine or Fosenopril* or Fosfenopril* or Fosinil* or Fosinopril* or Fosinorm or Fositen* or Fozitec or Gemopatril* or Gopten or Hiperlex or Idapril* or Idrapril* or Ilepatril* or Imidapril* or Indolapril* or Inhibace or Labopal or Libenzapril* or Lisidigal or Lisinopril* or Listril* or Lopirin or Lopril* or Lotensin or Lysinopril* or Mavik or Mixanpril* or Moex or Moexipril* or Monopril* or Moveltipril* or Newace or Nitrosocaptopril* or Novatec or Odrik or Omapatril* or Pentopril* or Perdix or Perindo* or Perstarium or Pirindopril* or Pivopril* or Prilace or Prinivil* or Quadropril* or Quinapril* or Ramace or Ramipril* or Ramitac or Ramiwin or Renitec or Renitek or Renormax or Renpress or Rentiapril* or Sampatril* or Spirapril* or Staril* or Tanatril* or Temocapril* or Tensocardil* or Teprotide or Trandolapril* or Triatec or Tritace or Udrik or Univasc or Utibapril* or Valine-Proline-Proline or Val-Pro-Pro or Valyl-Prolyl-Proline or Vanlev or Vasotec or Vesdil* or Xanef or Zabicipril* or Zabien or Zestril* or Zofenil* or Zofenopril* or Zofil* or Angiotensin Receptor Antagonist* or Angiotensin Receptor Block* or Angiotensin Receptor Inhibit* or Angiotensin II Receptor Antagonist* or Angiotensin II Receptor Block* or Angiotensin II Receptor Inhibit* or Angiotensin 2 Receptor Antagonist* or Angiotensin 2 Receptor Block* or Angiotensin 2 Receptor Inhibit* or Angiotensin 2 Type 1 Receptor Antagonist* or Angiotensin 2 Type 1 Receptor Block* or Angiotensin 2 Type 1 Receptor Inhibit* or Angiotensin II Type 1 Receptor Antagonist* or Angiotensin II Type 1 Receptor Block* or Angiotensin II Type 1 Receptor Inhibit* or Angiotensin 2 Type 2 Receptor Antagonist* or Angiotensin 2 Type 2 Receptor Block* or Angiotensin 2 Type 2 Receptor Inhibit* or Angiotensin II Type 2 Receptor Antagonist* or Angiotensin II Type 2 Receptor Block* or Angiotensin II Type 2 Receptor Inhibit* or Abitesartan or Allisartan or Aprovel or Atacand or Avapro or Azilsartan or Azor or Benicar or Candesartan or Cardosten or Cozaar or Diovan or Edarbi or Elisartan or Embusartan or Enoltasosartan or Eprosartan or Fimasartan or Fonsartan or Forasartan or Irbesartan or Kalpress or Karvea or Losartan or Micardis or Milfasartan or Miten or Nisis or Olmesartan or Olmetec or Olodanrigan or Omesartan or Pomisartan or Pratosartan or Pritor or Provas or Ripisartan or Saprisartan or Saralasin or Sartan* or Sparsentan or Tareg or Tasosartan or Telmisartan or Teveten or Tonlamarsen or Vals or Valsartan or Votum or Zolasartan (Abstract)

3: Randomized or Randomized or Placebo or Randomly or Trial or Groups (Title) OR Randomized or Randomized or Placebo or Randomly or Trial or Groups (Abstract)

4: Double-Blind* or Doubleblind* or Double-Mask* or Double-Dum* or Doubledum* or Triple-Blind* or Trippleblind* or Treble-Blind* or Trebleblind* or Quadruple-Blind* or Quadrupleblind* or Blind* or Mask* or Dumm* (Title) OR Double-Blind* or Doubleblind* or Double-Mask* or Double-Dum* or Doubledum* or Triple-Blind* or Trippleblind* or Treble-Blind* or Trebleblind* or Quadruple-Blind* or Quadrupleblind* or Blind* or Mask* or Dumm* (Abstract)

5: Mortalit* or "End Of Life" or Death* or Fatal* or Survival* OR Sudden Cardiac Arrest* or Died or Dead (Title) OR Mortalit* or "End Of Life" or Death* or Fatal* or Survival* OR Sudden Cardiac Arrest* or Died or Dead (Abstract)

6: #5 AND #4 AND #3 AND #2 AND #1

7: #5 AND #4 AND #3 AND #2 AND #1

**BOX 6. Search strategy for WHO ICTRP**

Advanced Search

Hypertension OR High Blood Pressure OR Coronary Artery Disease OR Heart Infarction OR Heart Failure OR Stroke OR Transient Ischemic Attack OR Brain Ischemia OR Brain Infarction OR Diabetes OR Heart Attack OR Chronic Kidney OR Chronic Renal OR Nephropathy **in the Condition**

Azilsartan OR Benazepril OR Candesartan OR Captopril OR Delapril OR Enalapril OR Eprosartan OR Fimasartan OR Fosinopril OR Fosinopril OR Imidapril OR Irbesartan OR Lisinopril OR Losartan OR Olmesartan OR Perindopril OR Quinapril OR Ramipril OR Telmisartan OR Trandolapril OR Valsartan OR Moexipril **in the Intervention**

**Recruitment status is** ALL

**Supplementary Table 2.** Risk of bias assessment. The table provides an assessment of the risk of bias in various clinical trials evaluating ACE inhibitors (ACE-Is) and angiotensin receptor blockers (ARBs). Each trial is scrutinized across seven domains: random sequence generation (selection bias), allocation concealment, blinding of participants and personnel (performance bias), blinding of outcome assessment (detection bias), incomplete outcome data (attribution bias), selective outcome reporting (reporting bias), and other biases.

| **Trials** | **Risk of bias (Low/ High/Unclear)** | | | | | | |  |
| --- | --- | --- | --- | --- | --- | --- | --- | --- |
|  | **DOMAINS** | | | | | | |  |
|  | **Random sequence generation (selection bias)** | **Allocation concealment (selection bias)** | **Blinding of participants and personnel (performance bias)** | **Blinding of outcome assessment (detection bias)** | **Incomplete outcome data (attribution bias)** | **Selective outcome reporting? (reporting bias)** | **Other bias** |  |
|  |  |  |  |  |  |  |  |  |
| **ACE-Is** | | | | | | | |  |
| DIAHBYCAR | Low | Unclear | Unclear | Low | Unclear | Low | Unclear |  |
| DREAM | Low | Low | Unclear | Low | Unclear | Low | Unclear |  |
| EUROPA | Unclear | Unclear | Low | Low | Unclear/low? | Low | Unclear |  |
| HOPE | Unclear | Unclear | Low | Low | Low | Low |  |  |
| PART-2 | Low | Unclear | Low | Unclear | Low | Low |  |  |
| PEACE | Unclear | Unclear | Low | Unclear | Low | Low |  |  |
| PREAMI | Low | Unclear | Low | Unclear | Low | Low |  |  |
| PROGRESS | Low | Low | Low | Low | Low | Low |  |  |
| QUIET | Low | Unclear | Low | Low | Low | Low |  |  |
| **ARBs** | | | | | | | |  |
| ACTIVE-I | Low | Low | Low | Low | Low | Low | Unclear |  |
| DIRECT Protect-2 | Low | Low | Low | Low | Low | Low |  |  |
| IDNT | High | Unclear | Low | Unclear | Low | Low |  |  |
| NAVIGATOR | Low | Unclear | Low | Low | Low | Low |  |  |
| RENAAL | Low | Unclear | Low | Low | Low | Low |  |  |
| ROADMAP | Low | Low | Low | Low | Low | Low |  |  |
| SCOPE | Low | Low | Low | Low | Unclear | Low |  |  |
| TRANSCED | Low | Low | Low | Low | Unclear | Low |  |  |

**Supplementary Table 3. Summary of the certainty of evidence based on the GRADE score**

| **Outcome** | **Intervention** | **Number of trials** | **Number of participants** | **HR (95% CI)** | **Assumed risk (per 1000)** | **Anticipated absolute effects** | **Certainty (GRADE)** | **Comments** |
| --- | --- | --- | --- | --- | --- | --- | --- | --- |
| *Cardiovascular mortality* | ACEi | 7 | 46951 | 0.88 (0.78 to 0.99) | 38 | 33.4 (from 29.6 to 37.6) | ⨁⨁⨁◯ | Downgraded for imprecision: CI near 1.0 and unclear bias in some studies. |
|  | ARB | 6 | 43165 | 1.03 (0.95 to 1.11) | 38 | 39.1 (from 36.1 to 42.2) | ⨁⨁◯◯ | Imprecision and no significant effect. |
|  | ACEi + ARB | 13 | 90116 | 0.95 (0.88 to 1.02) | 38 | 36.1 (from 33.4 to 38.8) | ⨁⨁◯◯ | CI crosses 1.0; downgraded for imprecision. |
| *All-cause mortality* | ACEi | 7 | 45968 | 0.92 (0.85 to 0.99) | 58 | 53.4 (from 49.3 to 57.4) | ⨁⨁⨁◯ | Borderline CI; some concerns for publication bias. |
|  | ARB | 6 | 45499 | 1.00 (0.94 to 1.06) | 58 | 58.0 (from 54.5 to 61.5) | ⨁⨁◯◯ | Neutral effect; downgraded for imprecision. |
|  | ACEi + ARB | 13 | 91467 | 0.96 (0.91 to 1.01) | 58 | 55.7 (from 52.8 to 58.6) | ⨁⨁◯◯ | Non-significant effect; downgraded for imprecision. |
| *MACE* | ACEi | 7 | 43725 | 0.90 (0.81 to 1.00) | 170 | 153.0 (from 137.7 to 170.0) | ⨁⨁◯◯ | Downgraded for inconsistency (I² > 60%) and imprecision. |
|  | ARB | 7 | 44684 | 0.94 (0.89 to 0.99) | 200 | 188.0 (from 178.0 to 198.0) | ⨁⨁⨁◯ | Moderate certainty: consistent and significant effect. |
|  | ACEi + ARB | 14 | 88345 | 0.92 (0.87 to 0.97) | 185 | 170.2 (from 160.9 to 179.4) | ⨁⨁⨁◯ | Consistent benefit with moderate certainty. |
| *Stroke* | ACEi | 6 | 42795 | 0.82 (0.71 to 0.96) | 43 | 35.3 (from 30.5 to 41.3) | ⨁⨁⨁◯ | Moderate heterogeneity; effect significant. |
|  | ARB | 7 | 42180 | 0.85 (0.77 to 0.94) | 47 | 39.9 (from 36.2 to 44.2) | ⨁⨁⨁◯ | Consistent effect with moderate heterogeneity. |
|  | ACEi + ARB | 13 | 84975 | 0.84 (0.77 to 0.91) | 45 | 37.8 (from 34.6 to 41.0) | ⨁⨁⨁◯ | Strong, consistent effect; low heterogeneity. |
| *Myocardial infarction* | ACEi | 6 | 42816 | 0.83 (0.73 to 0.94) | 43 | 35.7 (from 31.4 to 40.4) | ⨁⨁⨁◯ | Consistent effect; moderate heterogeneity. |
|  | ARB | 6 | 41815 | 0.91 (0.77 to 1.07) | 47 | 42.8 (from 36.2 to 50.3) | ⨁⨁◯◯ | Downgraded for imprecision; wide CI crossing 1. |
|  | ACEi + ARB | 12 | 84631 | 0.87 (0.78 to 0.96) | 45 | 39.1 (from 35.1 to 43.2) | ⨁⨁⨁◯ | Moderate certainty: consistent and significant. |
| *Incident heart failure* | ACEi | 7 | 43955 | 0.77 (0.69 to 0.85) | 49 | 37.7 (from 33.8 to 41.6) | ⨁⨁⨁⨁ | High certainty: consistent trials, narrow CI, low bias. |
|  | ARB | 6 | 41217 | 0.87 (0.76 to 1.00) | 54 | 47.0 (from 41.0 to 54.0) | ⨁⨁⨁◯ | Effect borderline significant; CI includes null. |
|  | ACEi + ARB | 13 | 85172 | 0.82 (0.75 to 0.89) | 52 | 42.6 (from 39.0 to 46.3) | ⨁⨁⨁⨁ | High certainty: low risk of bias, consistent effect. |

**SUPPLEMENTARY FIGURES**

**Supplementary figure 1.** Standardized pre-piloted data extraction form

**
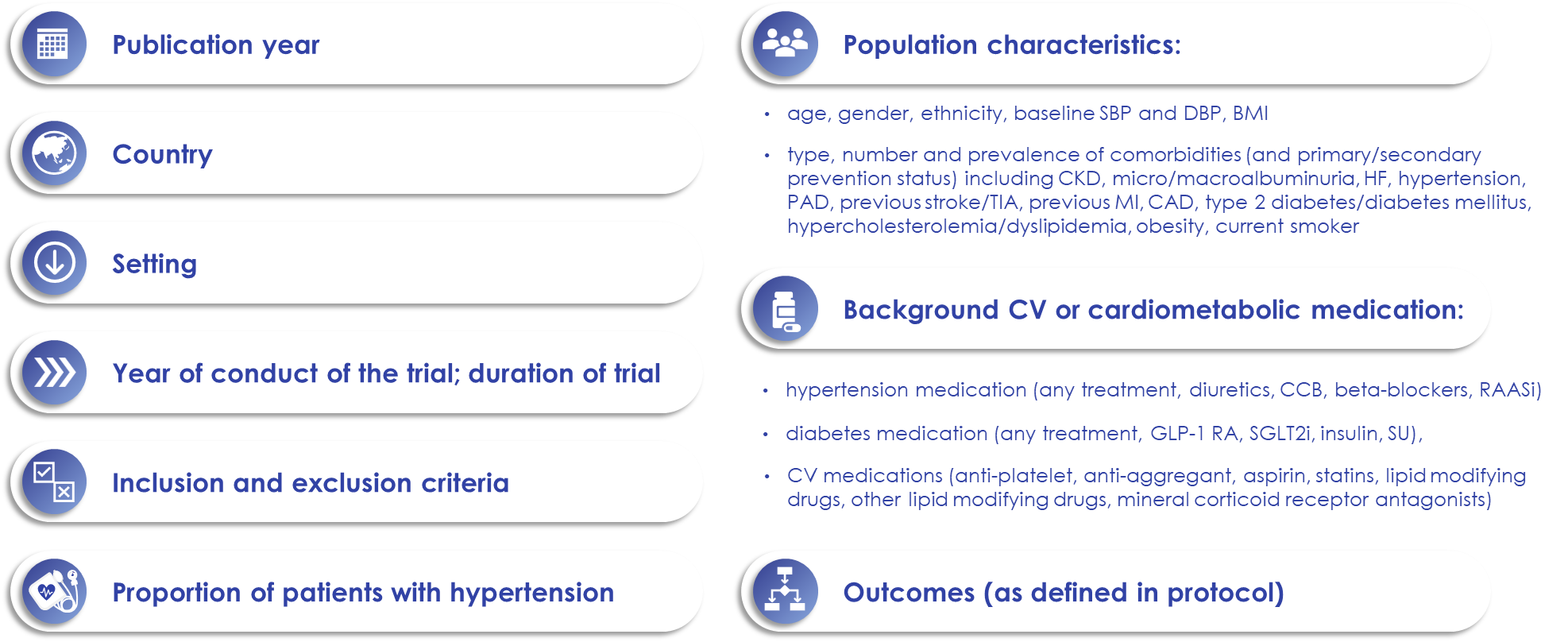
**

**Supplementary figure 2.** Difference in the rate of cardiovascular, all-cause mortality and major cardiovascular events (MACEs) in the placebo arm of the ACE-I and ARB trials. A) cardiovascular mortality event rate; B) All-cause mortality event rate; C) Major cardiovascular events (MACEs).

1. **Cardiovascular Mortality**

1. **All-cause mortality**


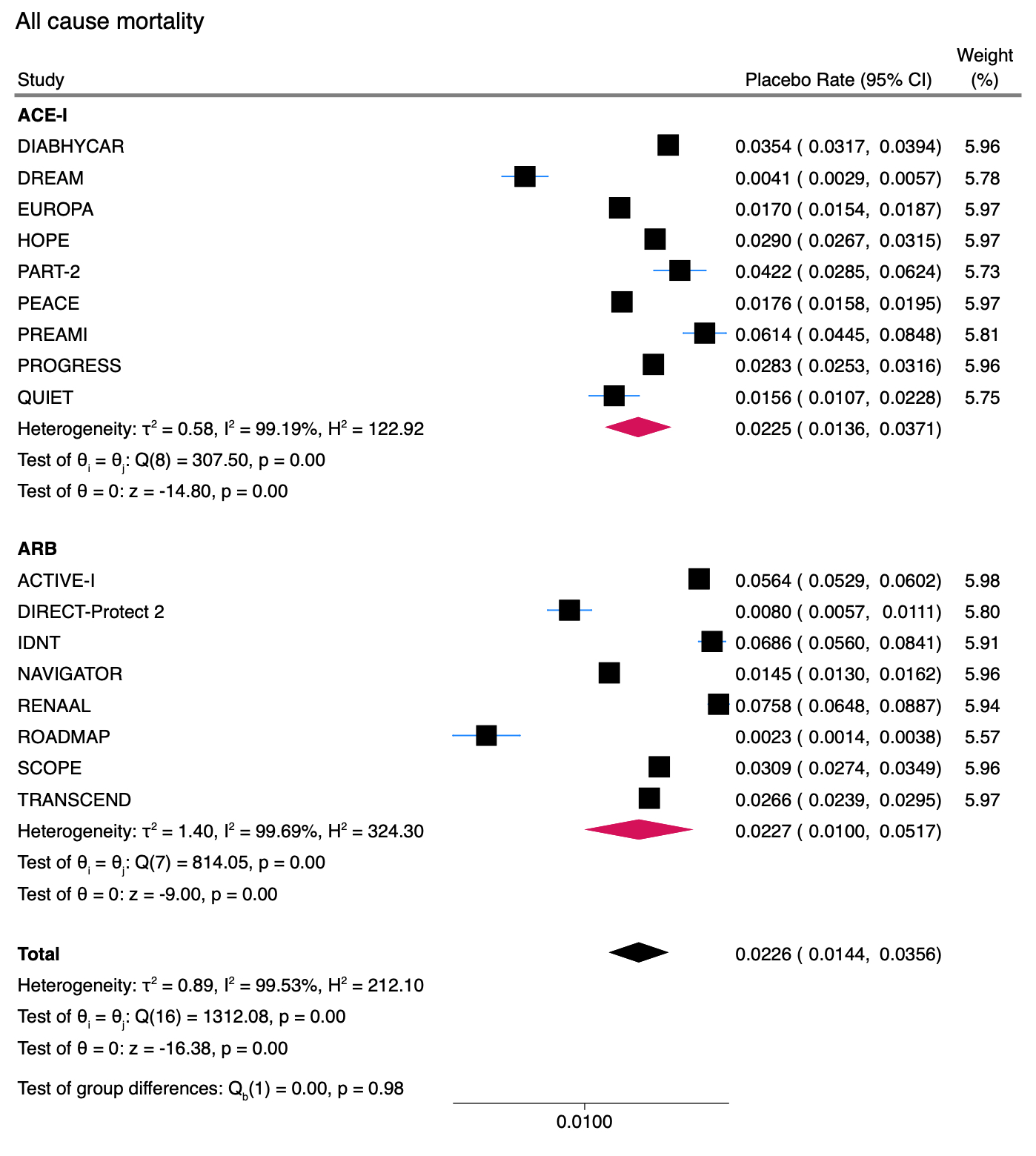


1. **Major Cardiovascular Events (MACEs)**

**Supplementary figure 3.** Results of the “leave-one-out” analysis. Single trials, one at a time, were excluded from the analysis to check if overall results could change following the single trial exclusion. The analysis was performed for A) cardiovascular mortality; B) all-cause mortality; C) heart failure; D) myocardial infarction; E) Stroke; F) major cardiovascular events (MACEs).

1. **Cardiovascular Mortality**

**ACEi**


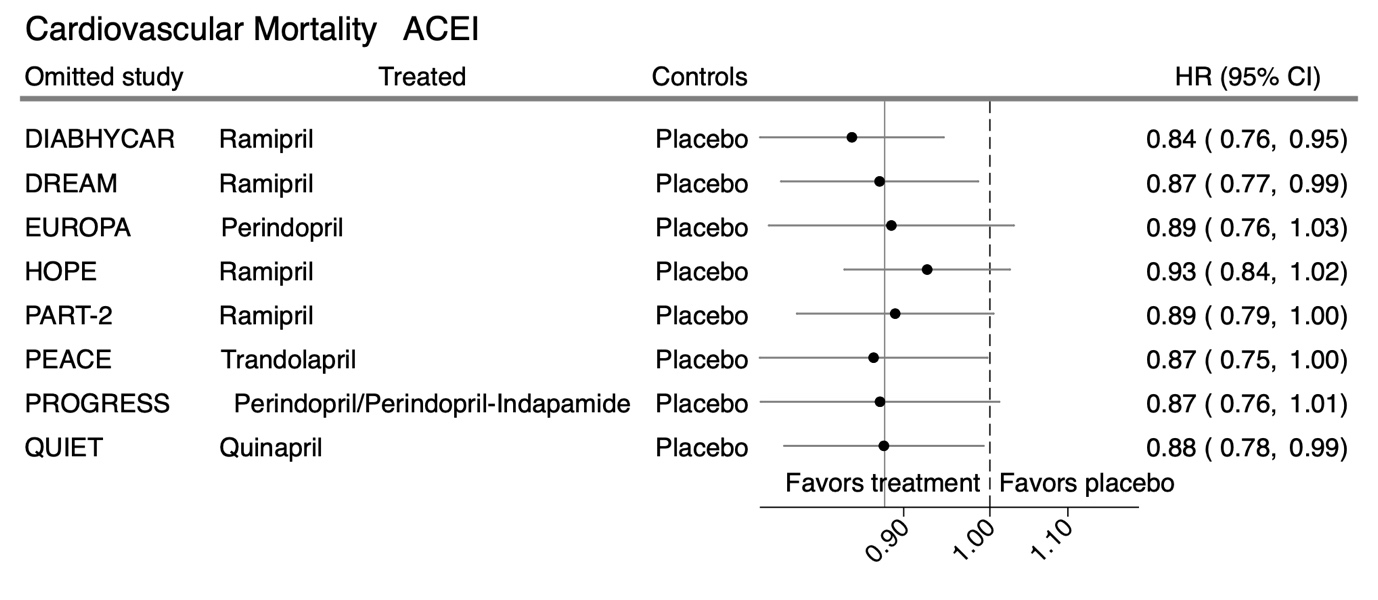


**ARBs**


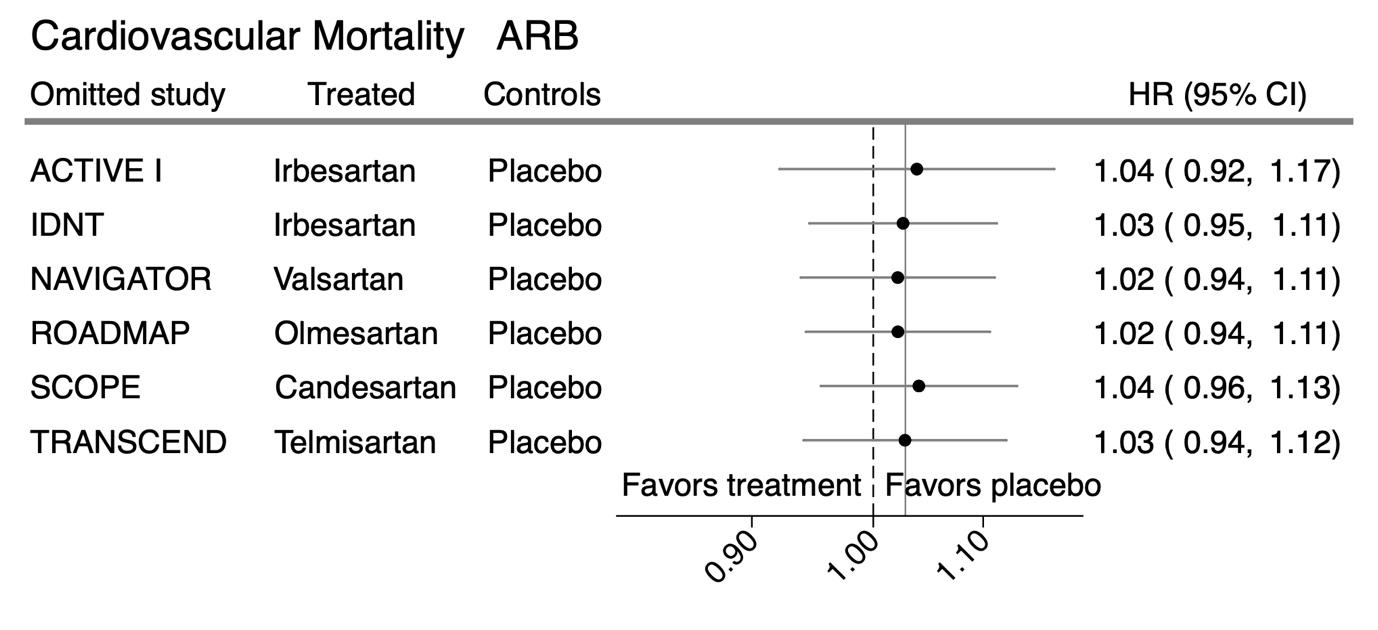


1. **All-cause Mortality**

**ACEi**


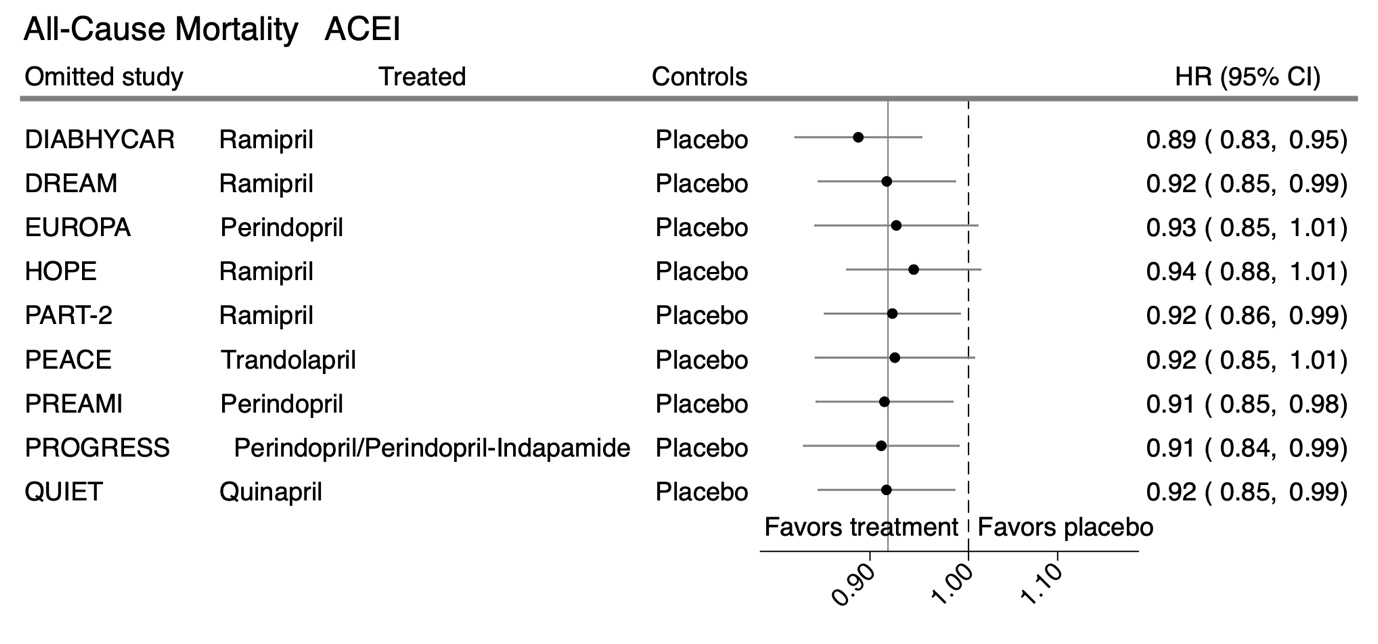


**ARBs**


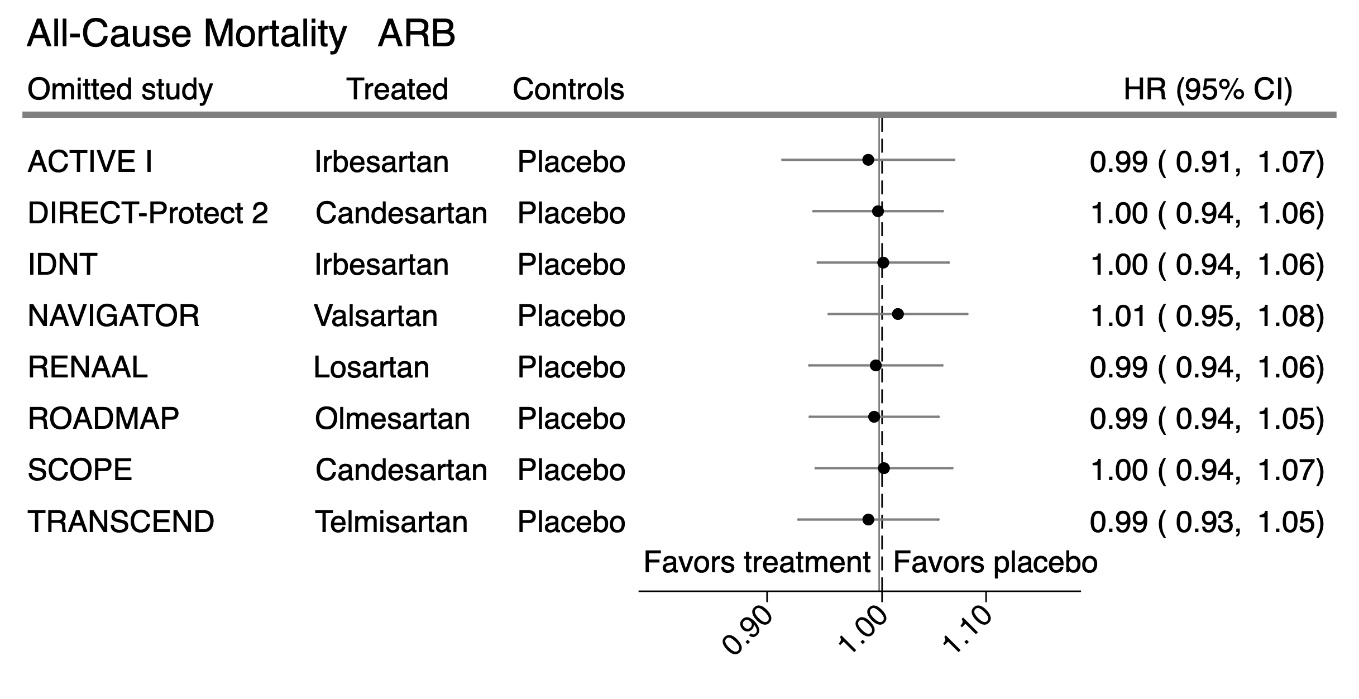


1. **Heart Failure**

**ACEi**


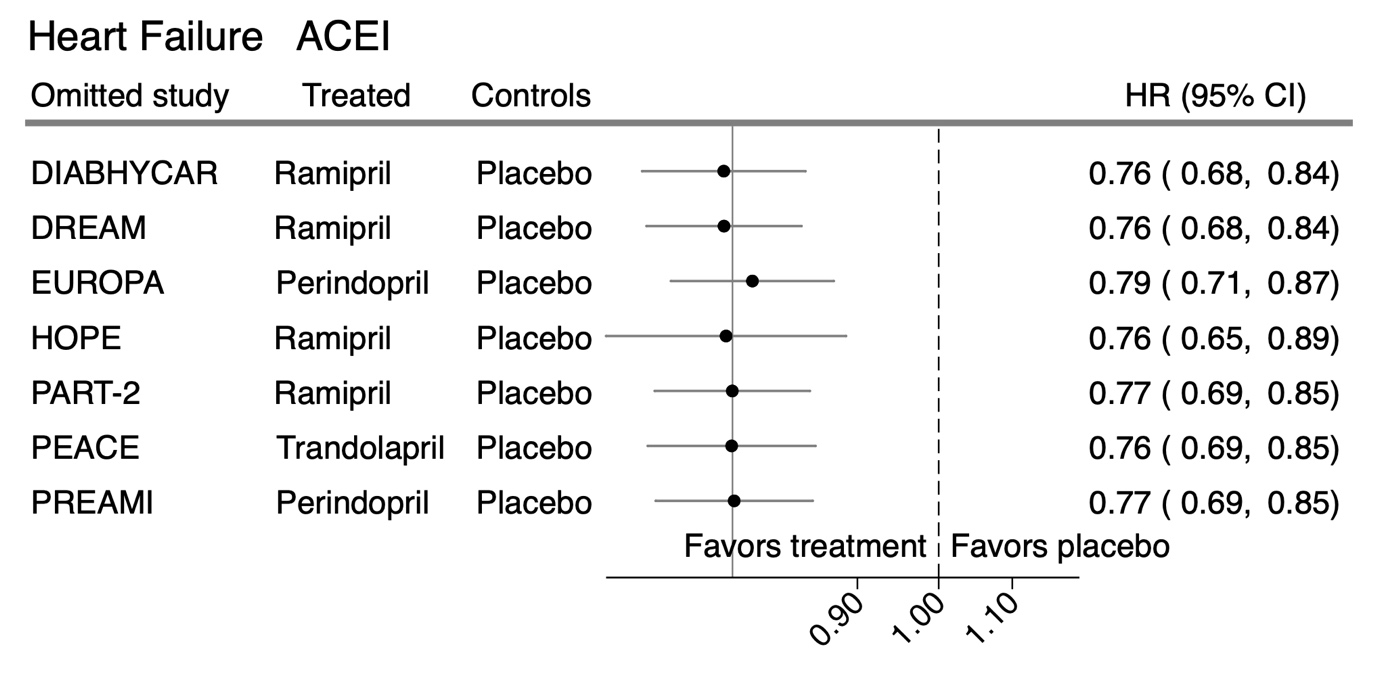


**ARBs**


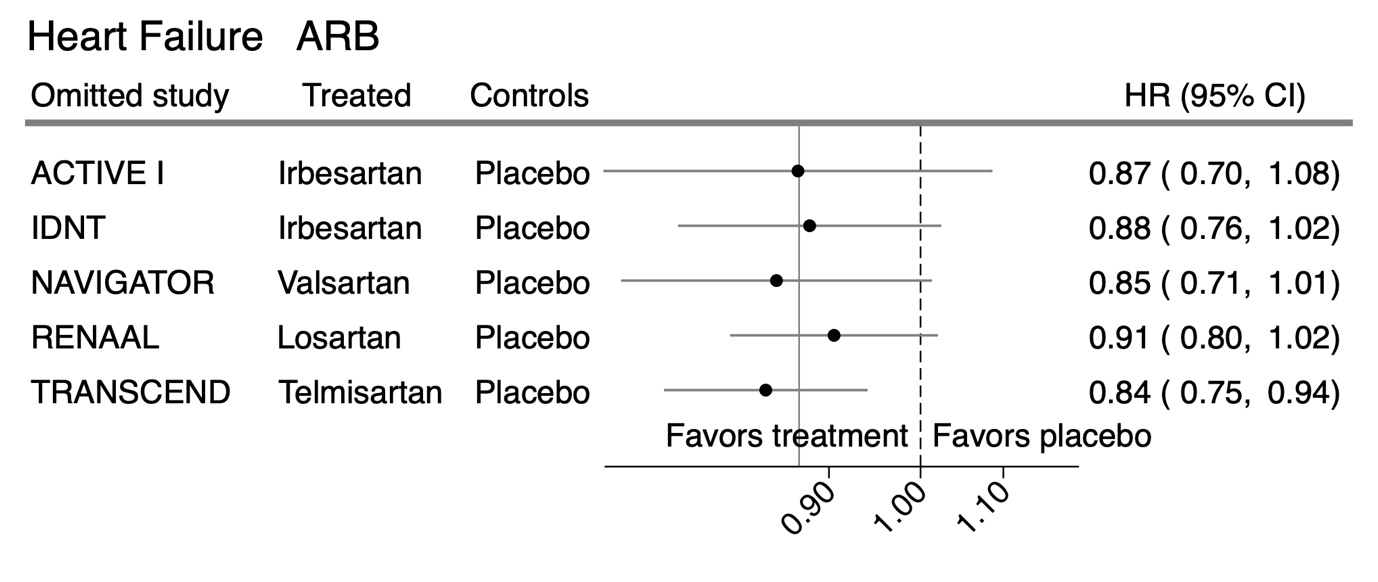


1. **Myocardial Infarction**

**ACEi**

**
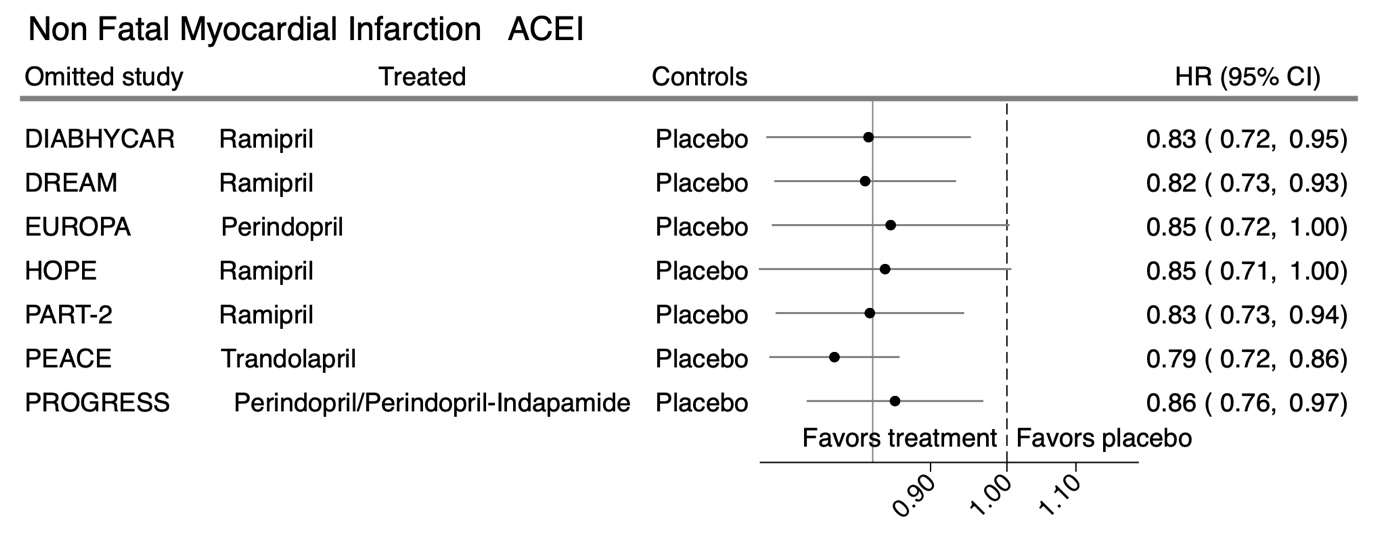
**

**ARBs**


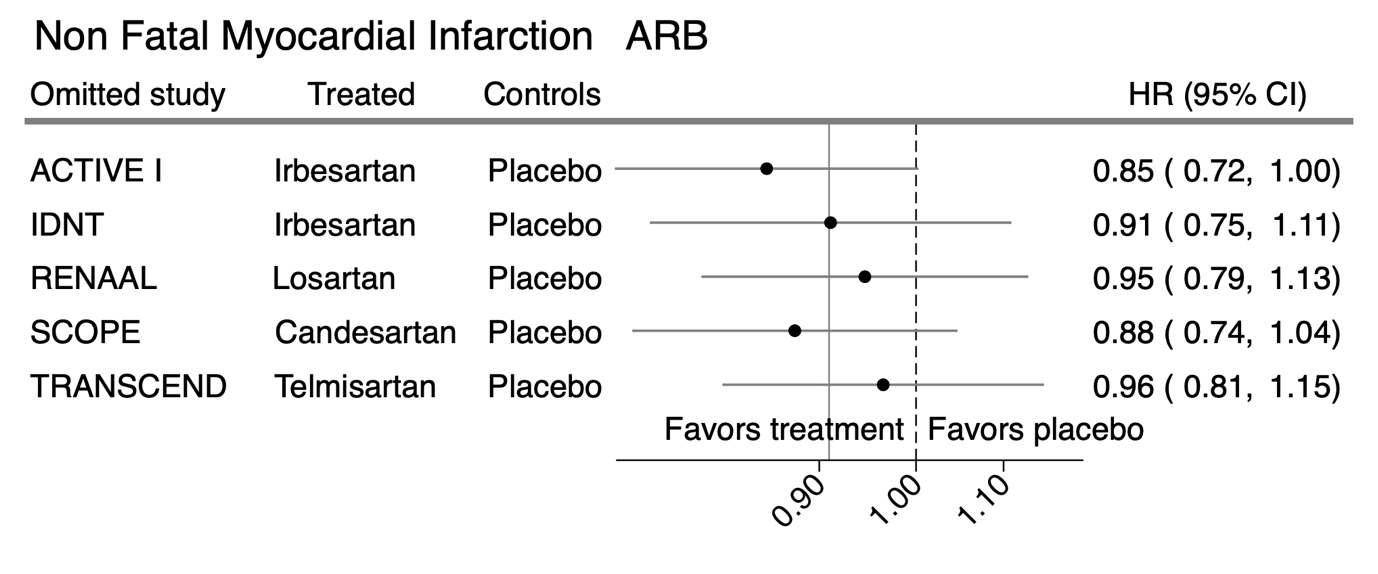


1. **Stroke**

**ACEi**

**
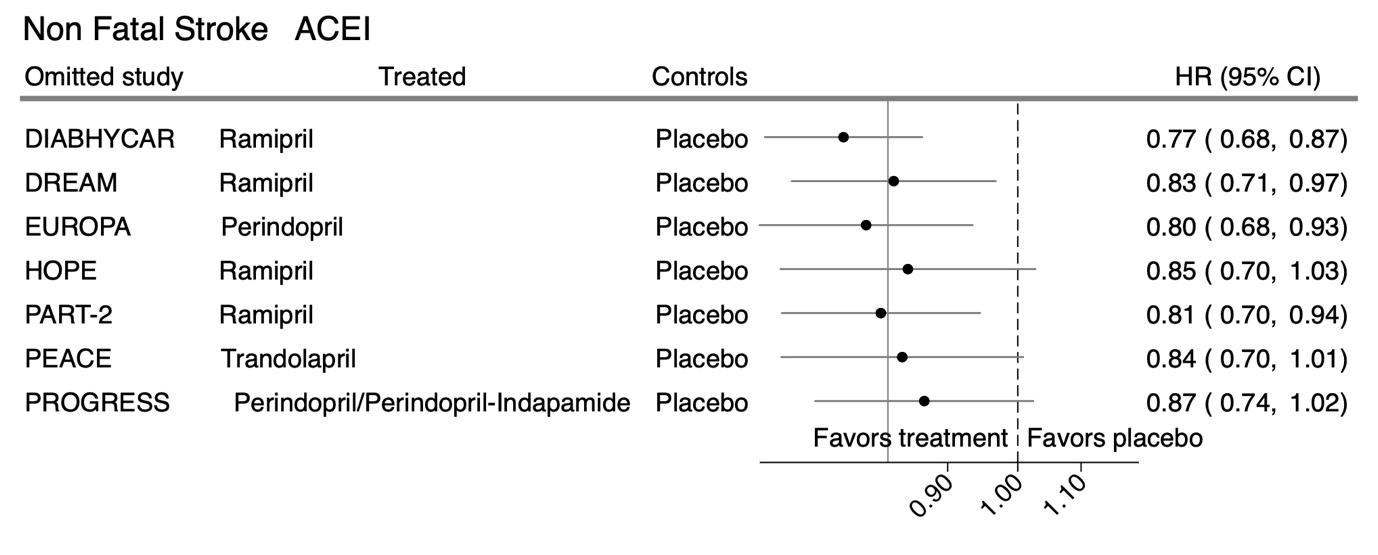
**

**ARBs**


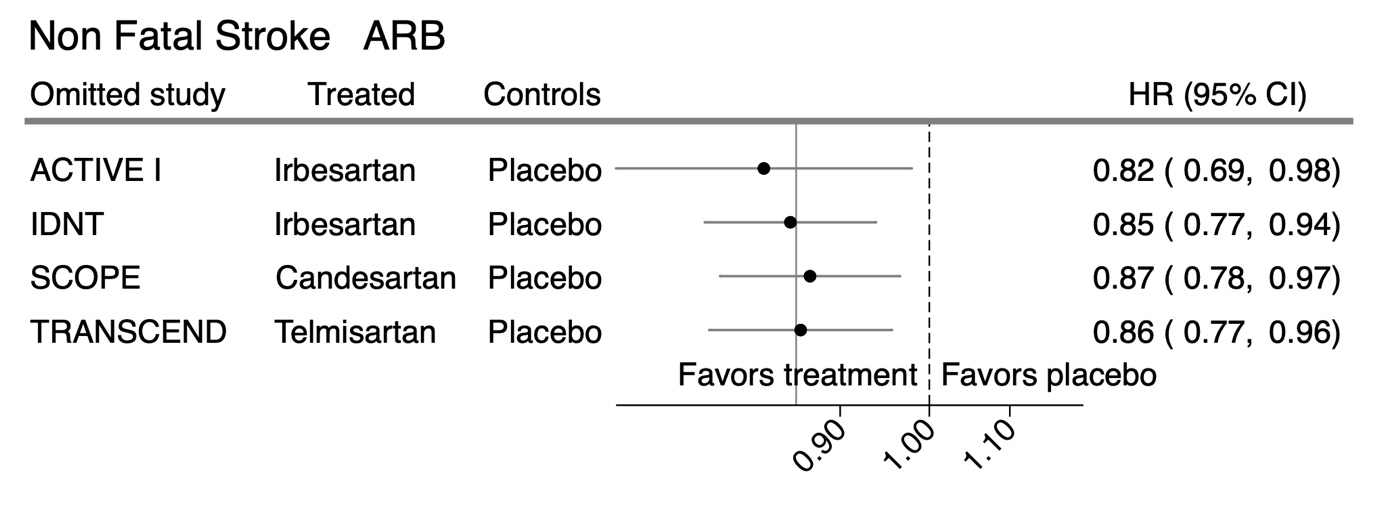


1. **Major Cardiovascular Events (MACEs)**

**ACEi**


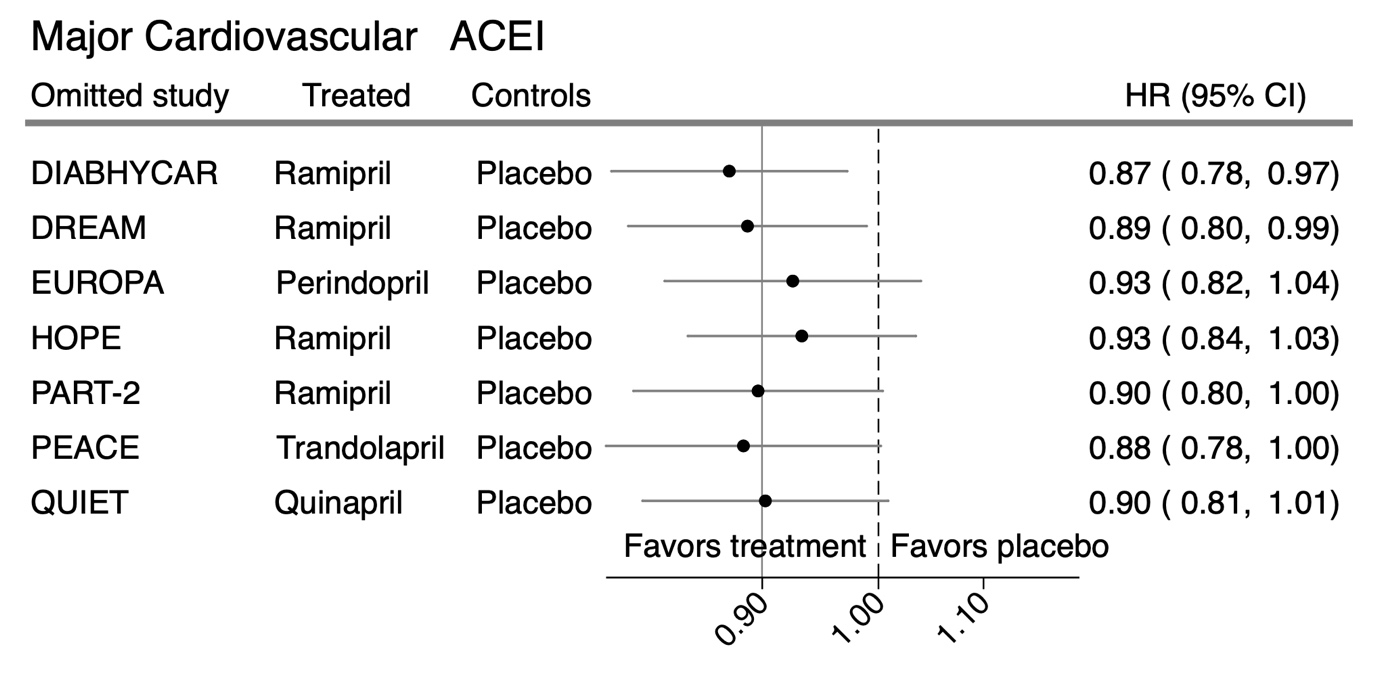


**ARBs**


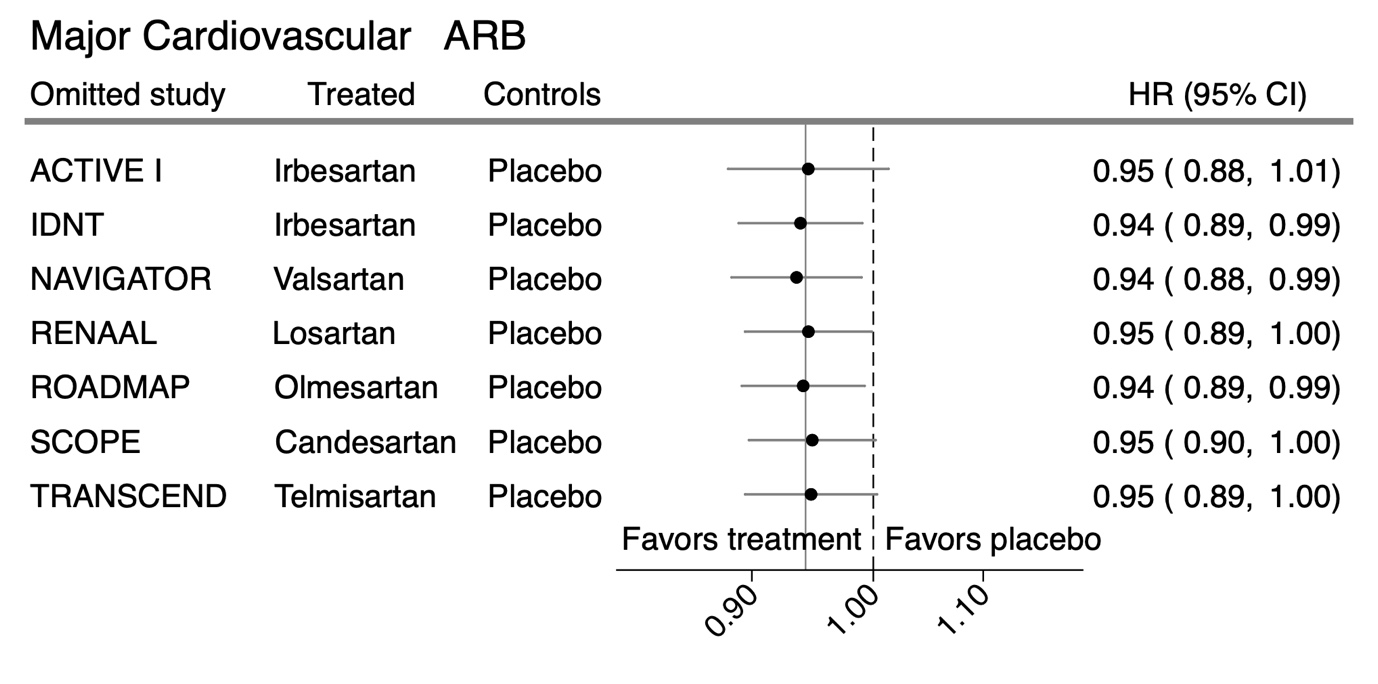

Supplement: oeag119_Supplementary_Data [file oeag119_supplementary_data.docx]
